# Validity Assessment of Self-reported Medication Use for Hypertension, Diabetes, and Dyslipidemia in a Pharmacoepidemiologic Study by Comparison With Health Insurance Claims

**DOI:** 10.2188/jea.JE20200089

**Published:** 2021-09-05

**Authors:** Minako Matsumoto, Sei Harada, Miho Iida, Suzuka Kato, Mizuki Sata, Aya Hirata, Kazuyo Kuwabara, Ayano Takeuchi, Daisuke Sugiyama, Tomonori Okamura, Toru Takebayashi

**Affiliations:** Department of Preventive Medicine and Public Health, School of Medicine, Keio University, Tokyo, Japan

**Keywords:** cohort-study, health insurance claims, medicines, self-report, validation

## Abstract

**Background:**

Although self-reported questionnaires are widely employed in epidemiologic studies, their validity has not been sufficiently assessed. The aim of this study was to evaluate the validity of a self-reported questionnaire on medication use by comparison with health insurance claims and to identify individual determinants of discordance in the Tsuruoka Metabolomics Cohort Study.

**Methods:**

Participants were 2,472 community-dwellers aged 37 to 78 years from the Tsuruoka Metabolomics Cohort Study. Information on lifestyle and medications was collected through a questionnaire. Sensitivity and specificity were determined using health insurance claims from November 2014 to March 2016, which were used as a standard. Potential determinants of discordance were assessed using multivariable logistic regression.

**Results:**

The self-reported questionnaire on medication use showed high validity. Sensitivity and specificity were 0.95 (95% CI, 0.93–0.96) and 0.97 (95% CI, 0.96–0.98) for antihypertensive medications, 0.94 (95% CI, 0.91–0.97) and 0.98 (95% CI, 0.98–0.99) for diabetes medications, and 0.84 (95% CI, 0.82–0.87) and 0.98 (95% CI, 0.97–0.99) for dyslipidemia medications, respectively. Males without high education and those who currently smoke cigarettes were found to be associated with discordant reporting which affected sensitivity, especially those with medication use for dyslipidemia.

**Conclusions:**

In this population-based cohort study, we found that the self-reported questionnaire on medication use was a valid measure to capture regular medication users. Sensitivity for dyslipidemia medications was lower than those for the other medications. Type of medication, sex, education years, and smoking status influenced discordance, which affected sensitivity in self-reporting.

## INTRODUCTION

The number of patients with three of the major lifestyle-related diseases—hypertension, diabetes and dyslipidemia—is increasing. These are major risk factors for cardiovascular disease.^1^^–^^3^ To assess relationships between risk factors and health outcomes in cohort studies, participant characteristics including medication use are often evaluated using self-reported questionnaire. Despite the possibility of information bias, however, the accuracy of self-reported questionnaires has not been sufficiently studied.^4^^,^^5^ In particular, few reports have explored the individual determinants of discordance between self-reported questionnaires on medication use and the true status of medication.

To date, only a few studies have evaluated the validity of self-reported medication use in population-based studies, and the results of these have been inconsistent.^6^^–^^8^ Although self-reported medication use for lifestyle-related disease has shown high validity with sensitivity over 70%, the sensitivity nevertheless varied from study to study. This inconsistency has been explained by differences in data collection method, type of medication, and surveyed populations. Moreover, only a few studies have identified individual determinants of discordance between self-reported medication use and true status of medication.^7^^,^^9^^,^^10^ These include sex,^7^^,^^9^ age,^7^^,^^10^ marital status,^9^ number of medications regularly taken,^10^ smoking status,^7^ health status^7^ and education years,^9^ albeit that the results varied among studies.

The aim of this study was to evaluate the validity of self-reported medication use for lifestyle-related diseases in our population-based Tsuruoka Metabolomics Cohort Study using health insurance claims as a standard. Individual determinants of discordance, such as social factors, were also examined.

## METHODS

### Japanese healthcare insurance system

Japan has a universal healthcare insurance system which covers all citizens.^11^ There are two types of coverage for individuals aged younger than 75 years, Employees’ Health Insurance and National Health Insurance (NHI). The former is managed by the workplace and covers salaried employees while the latter is managed by municipalities and covers individual proprietors, pensioners and those with irregular employment. On reaching 75 years of age, current NHI members are switched from NHI to Medical Care System for the Advanced Elderly. If an insured member goes to a hospital or pharmacy as an outpatient, their information is stored as health insurance claims data of medical/dental outpatient claims and pharmacy claims.

In Japan, long-term prescriptions are allowed, except for special medications, such as newly launched or psychoactive medications; newly launched medications, for example, can be prescribed in 2-week courses. In contrast, most medications, particularly those for lifestyle-related diseases, are prescribed in courses of 90 days duration or less.

### Study base

Participants of this study were 1,128 males and 1,344 females (total 2,472) who joined the follow-up survey of the Tsuruoka Metabolomics Cohort Study between April 2015 and March 2016 and those who were the beneficiaries of NHI and Medical Care System for the Advanced Elderly. Briefly, the Tsuruoka Metabolomics Cohort Study is a population-based study started in April 2012 in Tsuruoka City, Yamagata Prefecture, Japan. A total of 11,002 participants aged 35–74 years were recruited from municipal or worksite health check-ups in the city during the baseline period from 2012 to 2014 and enrolled. Follow-up surveys of this original cohort are conducted periodically. Participant information, including social factors, medical history, and medications was obtained from standardized self-administered questionnaires with face-to-face interview during the health check-up. Other measurements (height, weight, blood pressure, and laboratory data) were also collected during the check-up. All data were recorded using anonymized participant linkers. Details have been reported previously.^12^^–^^15^

The study was approved by the Medical Ethics Committee of the School of Medicine, Keio University, Tokyo, Japan (Approval No 20110264). All individual participants in this study provided written informed consent.

### Self-reported medication use

All participants were asked to complete a standardized self-administered questionnaire which included the items listed below (eMaterials 1). The answers were checked twice by interviewers using face-to-face interview.

• Are you currently (at least once a week) taking any medications? (yes or no).
[1] Medication for hypertension (yes or blank).
[2] Medication for blood sugar level-lowering (diabetes) (yes or blank).
[3] Medication for cholesterol-lowering (dyslipidemia) (yes or blank).

We defined participants who answered ‘yes’ to the first question as self-reported medication users and those who answered ‘no’ as non-users. Self-reported medication users who chose “Medication for hypertension”, “Medication for blood sugar level-lowering (diabetes)” or “Medication for cholesterol-lowering (dyslipidemia)” were defined as self-reported medication users against each disease.

### Medication use information from medical and pharmacy health insurance claims

Regular medication users were captured by using health insurance claims from October 2014 to March 2016 provided by Tsuruoka City.

To define medication categories, we used the drug database in Japan^16^^,^^17^ and codes of the Anatomical Therapeutic Chemical (ATC) provided by World Health Organization.^18^ For some medications which did not have an ATC code, we assigned the closest minimum code based on medication category. We defined antihypertensive medications as follows: medications with an ATC code starting from C02 or listed as a medication for hypertension in Japan (eTable 1). Medications for diabetes were as follows: medications with an ATC code starting from A10 or listed as a medication for diabetes in Japan (eTable 2). Medications for dyslipidemia were as follows: medications with an ATC code starting from C10 or listed as a medication for dyslipidemia in Japan (eTable 3).

As long-term prescriptions are allowed in Japan, even if the participants were not prescribed the medications during the survey month, they might take the medications that have been prescribed during the previous month. As a previous study observed that period of time shorter than 90 days are less sensitive to detect the medication use,^19^ we used two different time periods (3- and 6-month fixed time windows). The definition of 3-month fixed time window is the period of time that includes the survey month as the participants answered the self-reported questionnaires and the previous 2 months. The definition of 6-month fixed time window is the period of time that includes the survey month as the participants answered the self-reported questionnaires and the previous 5 months. Therefore, we identified ‘Regular medication users’ by collecting data for medications using 3- and 6-month fixed time windows and if the medications were prescribed during the period of time at least one time, we considered them as ‘regular medication users’ from an objective perspective.

### Additional covariate data of sociodemographic information

Marital status was classified as married if a participant answered ‘yes’ to the question ‘Do you currently have a spouse? (even if not living together)’. If a participant answered ‘no’, they were classified as single, divorced or widowed. If a participant’s last education status was an elementary school, junior high school or high school, we classified them as having 12 or fewer years of education years. If they had graduated from a technical college, junior college, university or graduate school, we classified them as having more than 12 years of education years. Job status was classified as ‘currently working’ if participants were not homemakers or unemployed. We defined the current smokers as those who smoked cigarettes currently and current drinkers as those who consumed alcohol more than 20 g every day. Those who maintained the habit of moderate exercising at least 30 minutes more than two times per week and kept the habit for more than 1 year were defined as regular exercisers. The information was collected at the baseline survey and updated at the follow-up survey if their status had changed.

### Statistical methods

We analyzed 2,472 beneficiaries (1,128 males and 1,344 females) of NHI or Medical Care System for the Advanced Elderly in this study because data on Employees’ Health Insurance beneficiaries was not available at this time. Differences between males and females were determined by using Student’s *t*-test for continuous variables and Chi-square test for categorized variables.

We evaluated the prevalence of medication use as determined by the standardized self-administered questionnaire and by the health insurance claims separately. To assess the validity of self-reported medication use, we used the health insurance claims as a standard. Sensitivity, specificity, and agreement were calculated with 95% confidence intervals (CIs). Sensitivity identifies the proportion of self-reported medication users among regular medication users, while specificity identifies the proportion of non-users according to the questionnaire among non-users detected by the health insurance claims. Agreement between self-reported medication use and the health insurance claims was calculated using the kappa statistics. The kappa statistics vary from 0 to 1 and are interpreted as follows: fair to poor (<0.40), moderate (0.41–0.60), substantial (0.61–0.80), and almost perfect (>0.81).^20^^,^^21^

Furthermore, we performed logistic regression analysis to examine potential determinants of discordance which affected sensitivity in each medication group, such as sex, age, marital status, education years, job status, smoking status, drinking status and regular exercise habit. Odds ratios (ORs) with 95% CIs were calculated. Multivariable logistic regression was performed in each medication group by adjusting for all potential determinants mentioned above. Subgroup analyses stratified by concurrent therapeutic areas, sex, education years, and smoking status were also performed. Also, we performed logistic regression analysis to examine potential determinants of discordance, which affected not only sensitivity but also specificity in each medication group. *P* < 0.05 was considered statistically significant.

All statistical analyses were performed using SAS 9.4 (SAS Institute Inc., Cary, NC, USA).

## RESULTS

### Basic characteristics

Table 1 shows the characteristics of participants. Mean and standard deviation (SD) age was 66 (SD, 6.9) years in total, and 65 (SD, 7.5) in males and 66 (SD, 6.5) in females. A higher proportion of males were married, working, current smokers, current drinkers, or taking prescribed medications for hypertension or diabetes than females. The most commonly prescribed medications were antihypertensive medications. With a 3-month fixed time window, the proportion of participants who took antihypertensive medications was 39.0% (males 43.7% and females 35.0%), versus dyslipidemia medications at 30.8% (males 24.6% and females 36.0%) and diabetes medications at 9.1% (males 12.1% and females 6.5%).

**Table 1.** Characteristics of the study population

	Total		Males		Females		

	*N*	% or SD	*N*	% or SD	*N*	% or SD	*P*-value^a^
***N***	2,472		1,128		1,344		
**Age, years^b^**	66	6.9	65	7.5	66	6.5	0.0331
**Married, Yes**	2,057	83.2%	998	88.5%	1,059	78.8%	<0.0001
**More than 12 years of education, Yes**	509	20.6%	237	21.0%	272	20.2%	<0.0001
**Currently working, Yes**	1,502	60.8%	804	71.3%	698	51.9%	<0.0001
**Current smoker, Yes**	305	12.3%	277	24.6%	28	2.1%	<0.0001
**Current drinker, Yes**	539	21.8%	489	43.4%	50	3.7%	<0.0001
**Regular exerciser, Yes**	828	33.5%	401	35.5%	427	31.8%	0.0319
**Medication prevalence according to self-report**							
Antihypertensive medication	957	38.7%	485	43.0%	472	35.1%	<0.0001
Diabetes medication	245	9.9%	149	13.2%	96	7.1%	<0.0001
Dyslipidemia medication	676	27.3%	211	18.7%	465	34.6%	<0.0001
**Medication prevalence according to self-report by concurrent therapeutic areas**							
Antihypertensive medication	527	21.3%	303	26.9%	224	16.7%	<0.0001
Diabetes medication	59	2.4%	44	3.9%	15	1.1%	<0.0001
Dyslipidemia medication	278	11.2%	61	5.4%	217	16.1%	<0.0001
Antihypertensive + Diabetes medications	72	2.9%	48	4.3%	24	1.8%	0.0003
Antihypertensive + Dyslipidemia medications	284	11.5%	93	8.2%	191	14.2%	<0.0001
Diabetes + Dyslipidemia medications	40	1.6%	16	1.4%	24	1.8%	0.4710
Antihypertensive + Diabetes + Dyslipidemia medications	74	3.0%	41	3.6%	33	2.5%	0.0865
**3-month fixed time window**							
Antihypertensive medication	963	39.0%	493	43.7%	470	35.0%	<0.0001
Diabetes medication	225	9.1%	137	12.1%	88	6.5%	<0.0001
Dyslipidemia medication	762	30.8%	278	24.6%	484	36.0%	<0.0001
**Three-month fixed time window by concurrent therapeutic areas**							
Antihypertensive medication	470	19.0%	263	23.3%	207	15.4%	<0.0001
Diabetes medication	36	1.5%	26	2.3%	10	0.7%	0.0013
Dyslipidemia medication	289	11.7%	70	6.2%	219	16.3%	<0.0001
Antihypertensive + Diabetes medications	64	2.6%	44	3.9%	20	1.5%	0.0002
Antihypertensive + Dyslipidemia medications	348	14.1%	141	12.5%	207	15.4%	0.0388
Diabetes + Dyslipidemia medications	44	1.8%	22	2.0%	22	1.6%	0.5572
Antihypertensive + Diabetes + Dyslipidemia medications	81	3.3%	45	4.0%	36	2.7%	0.0682
**Six-month fixed time window**							
Antihypertensive medication	975	39.4%	497	44.1%	478	35.6%	<0.0001
Diabetes medication	231	9.3%	141	12.5%	90	6.7%	<0.0001
Dyslipidemia medication	778	31.5%	286	25.4%	492	36.6%	<0.0001
**Six-month fixed time window by concurrent therapeutic areas**							
Antihypertensive medication	473	19.1%	264	23.4%	209	15.6%	<0.0001
Diabetes medication	37	1.5%	27	2.4%	10	0.7%	0.0008
Dyslipidemia medication	294	11.9%	75	6.6%	219	16.3%	<0.0001
Antihypertensive + Diabetes medications	64	2.6%	45	4.0%	19	1.4%	<0.0001
Antihypertensive + Dyslipidemia medications	354	14.3%	142	12.6%	212	15.8%	0.0243
Diabetes + Dyslipidemia medications	46	1.9%	23	2.0%	23	1.7%	0.5482
Antihypertensive + Diabetes + Dyslipidemia medications	84	3.4%	46	4.1%	38	2.8%	0.0874

SD, standard deviation.

^a^*P*-values for differences between males and females were determined by using Student’s *t*-test for continuous variables and Chi-square test for categorized variables.

^b^Reported as mean.

### Validity of self-reported medication use

Validation was performed between medication use from a self-reported questionnaire and health insurance claims (Table 2). We also conducted the same analyses stratified by sex (data not shown) and concurrent therapeutic areas (eTable 4). Although there were no obvious differences in sensitivity, specificity or kappa scores between 3- and 6-month fixed time windows, we used the 3-month fixed time window for the following analyses as it showed slightly higher sensitivity than the 6-month window.

**Table 2.** Validity of self-reported medication use

	Three-month fixed time window			Six-month fixed time window		
	
	Antihypertensive medication	Diabetes medication	Dyslipidemia medication	Antihypertensive medication	Diabetes medication	Dyslipidemia medication
True-positive, *N*	913	211	643	918	213	654
True-negative, *N*	1,465	2,213	1,677	1,458	2,209	1,672
False-positive, *N*	44	34	33	39	32	22
False-negative, *N*	50	14	119	57	18	124
Sensitivity (95% CI)	0.95 (0.93–0.96)	0.94 (0.91–0.97)	0.84 (0.82–0.87)	0.94 (0.93–0.96)	0.92 (0.89–0.96)	0.84 (0.81–0.87)
Specificity (95% CI)	0.97 (0.96–0.98)	0.98 (0.98–0.99)	0.98 (0.97–0.99)	0.97 (0.97–0.98)	0.99 (0.98–0.99)	0.99 (0.98–0.99)
Kappa score (95% CI)	0.92 (0.90–0.94)	0.89 (0.86–0.92)	0.85 (0.83–0.87)	0.92 (0.90–0.93)	0.88 (0.85–0.92)	0.86 (0.84–0.88)

CI, confidence interval.

The self-reported use of antihypertensive medications and diabetes medications predicted the regular use with high sensitivity (3-month fixed time window, 0.95 for antihypertensive medications and 0.94 for diabetes medications; 6-month fixed time window, 0.94 for antihypertensive medications and 0.92 for diabetes medications). In contrast, the self-reported use for dyslipidemia medications showed lower sensitivity (3-month fixed time window, 0.84; 6-month fixed time window, 0.84) than those for the other medications. Specificities were all over 0.97. Also, agreement of dyslipidemia medications was lower than those for the other medications, but still within the almost perfect kappa scores (3-month fixed time window, 0.85; 6-month fixed time window, 0.86). Sensitivity was better in one category than two or three categories of therapeutic areas.

### Determinants of discordance

Analyses of subgroups with the 3-month fixed time window stratified by sociodemographic factors including sex, age, marital status, education years, job status, smoking status, drinking status, and regular exercise habit are shown in Table 3. In the antihypertensive medications and the diabetes medications groups, sensitivity and specificity were all over 0.88 and kappa scores were all over 0.82 regardless of sociodemographic factors.

**Table 3.** Validity of self-reported medication use among subgroups with a three-month fixed time window

	Sex		Age		Married	
		
	Males	Females	65–78 years	35–64 years	Yes	No
**Antihypertensive medication**						
True-positive, *N*	465	448	668	245	766	141
True-negative, *N*	615	850	777	688	1,212	244
False-positive, *N*	20	24	35	9	36	8
False-negative, *N*	28	22	40	10	43	6
Sensitivity (95% CI)	0.94 (0.92–0.96)	0.95 (0.93–0.97)	0.94 (0.93–0.96)	0.96 (0.94–0.98)	0.95 (0.93–0.96)	0.96 (0.93–0.99)
Specificity (95% CI)	0.97 (0.95–0.98)	0.97 (0.96–0.98)	0.96 (0.94–0.97)	0.99 (0.98–1.00)	0.97 (0.96–0.98)	0.97 (0.95–0.99)
Kappa score (95% CI)	0.91 (0.89–0.94)	0.92 (0.90–0.95)	0.90 (0.88–0.92)	0.95 (0.93–0.97)	0.92 (0.90–0.94)	0.92 (0.89–0.96)
**Diabetes medication**						
True-positive, *N*	126	85	150	61	179	29
True-negative, *N*	968	1,245	1,333	880	1,840	362
False-positive, *N*	23	11	27	7	27	6
False-negative, *N*	11	3	10	4	11	2
Sensitivity (95% CI)	0.92 (0.87–0.97)	0.97 (0.93–1.00)	0.94 (0.90–0.98)	0.94 (0.88–1.00)	0.94 (0.91–0.98)	0.94 (0.85–1.02)
Specificity (95% CI)	0.98 (0.97–0.99)	0.99 (0.99–1.00)	0.98 (0.97–0.99)	0.99 (0.98–1.00)	0.99 (0.98–0.99)	0.98 (0.97–1.00)
Kappa score (95% CI)	0.86 (0.82–0.91)	0.92 (0.88–0.96)	0.87 (0.82–0.91)	0.91 (0.86–0.96)	0.89 (0.86–0.93)	0.87 (0.78–0.96)
**Dyslipidemia medication**						
True-positive, *N*	197	446	471	172	522	115
True-negative, *N*	836	841	935	742	1,407	261
False-positive, *N*	14	19	25	8	27	6
False-negative, *N*	81	38	89	30	101	17
Sensitivity (95% CI)	0.71 (0.66–0.76)	0.92 (0.90–0.95)	0.84 (0.81–0.87)	0.85 (0.80–0.90)	0.84 (0.81–0.87)	0.87 (0.81–0.93)
Specificity (95% CI)	0.98 (0.97–0.99)	0.98 (0.97–0.99)	0.97 (0.96–0.98)	0.99 (0.98–1.00)	0.98 (0.97–0.99)	0.98 (0.96–1.00)
Kappa score (95% CI)	0.75 (0.71–0.80)	0.91 (0.88–0.93)	0.83 (0.81–0.86)	0.88 (0.84–0.91)	0.85 (0.82–0.87)	0.87 (0.81–0.92)

	More than 12 years education		Currently working		Current smoker	
		
	Yes	No	Yes	No	Yes	No
**Antihypertensive medication**						
True-positive, *N*	171	739	499	412	86	821
True-negative, *N*	330	1,127	953	503	212	1,247
False-positive, *N*	6	37	23	21	3	41
False-negative, *N*	2	47	27	22	4	46
Sensitivity (95% CI)	0.99 (0.97–1.00)	0.94 (0.92–0.96)	0.95 (0.93–0.97)	0.95 (0.93–0.97)	0.96 (0.91–1.00)	0.95 (0.93–0.96)
Specificity (95% CI)	0.98 (0.97–1.00)	0.97 (0.96–0.98)	0.98 (0.97–0.99)	0.96 (0.94–0.98)	0.99 (0.97–1.00)	0.97 (0.96–0.98)
Kappa score (95% CI)	0.97 (0.94–0.99)	0.91 (0.89–0.93)	0.93 (0.91–0.95)	0.91 (0.88–0.94)	0.94 (0.90–0.99)	0.92 (0.90–0.93)
**Diabetes medication**						
True-positive, *N*	28	180	126	83	23	188
True-negative, *N*	474	1,730	1,353	851	275	1,926
False-positive, *N*	4	29	15	18	4	30
False-negative, *N*	3	11	8	6	3	11
Sensitivity (95% CI)	0.90 (0.80–1.01)	0.94 (0.91–0.98)	0.94 (0.90–0.98)	0.93 (0.88–0.98)	0.88 (0.76–1.01)	0.94 (0.91–0.98)
Specificity (95% CI)	0.99 (0.98–1.00)	0.98 (0.98–0.99)	0.99 (0.98–0.99)	0.98 (0.97–0.99)	0.99 (0.97–1.00)	0.98 (0.98–0.99)
Kappa score (95% CI)	0.88 (0.79–0.97)	0.89 (0.85–0.92)	0.91 (0.87–0.95)	0.86 (0.80–0.91)	0.86 (0.75–0.96)	0.89 (0.86–0.92)
**Dyslipidemia medication**						
True-positive, *N*	122	518	327	313	27	614
True-negative, *N*	365	1,302	1,090	578	258	1,409
False-positive, *N*	10	23	16	17	3	30
False-negative, *N*	12	107	69	50	17	102
Sensitivity (95% CI)	0.91 (0.86–0.96)	0.83 (0.80–0.86)	0.83 (0.79–0.86)	0.86 (0.83–0.90)	0.61 (0.47–0.76)	0.86 (0.83–0.88)
Specificity (95% CI)	0.97 (0.96–0.99)	0.98 (0.98–0.99)	0.99 (0.98–0.99)	0.97 (0.96–0.98)	0.99 (0.98–1.00)	0.98 (0.97–0.99)
Kappa score (95% CI)	0.89 (0.84–0.93)	0.84 (0.82–0.87)	0.85 (0.82–0.88)	0.85 (0.81–0.88)	0.69 (0.57–0.82)	0.86 (0.84–0.88)

	Current drinker		Regular exerciser	
	
	Yes	No	Yes	No
**Antihypertensive medication**				
True-positive, *N*	247	664	315	596
True-negative, *N*	279	1,179	479	292
False-positive, *N*	5	36	14	27
False-negative, *N*	8	42	20	30
Sensitivity (95% CI)	0.97 (0.95–0.99)	0.94 (0.92–0.96)	0.94 (0.91–0.97)	0.95 (0.94–0.97)
Specificity (95% CI)	0.98 (0.97–1.00)	0.97 (0.96–0.98)	0.97 (0.96–0.99)	0.97 (0.96–0.98)
Kappa score (95% CI)	0.95 (0.93–0.98)	0.91 (0.89–0.93)	0.91 (0.87–0.94)	0.93 (0.91–0.94)
**Diabetes medication**				
True-positive, *N*	47	163	54	156
True-negative, *N*	479	1,724	752	1,451
False-positive, *N*	10	23	15	18
False-negative, *N*	3	11	7	7
Sensitivity (95% CI)	0.94 (0.87–1.01)	0.94 (0.90–0.97)	0.89 (0.81–0.97)	0.96 (0.93–0.99)
Specificity (95% CI)	0.98 (0.97–0.99)	0.99 (0.98–0.99)	0.98 (0.97–0.99)	0.99 (0.98–0.99)
Kappa score (95% CI)	0.87 (0.79–0.94)	0.90 (0.86–0.93)	0.82 (0.74–0.89)	0.92 (0.89–0.95)
**Dyslipidemia medication**				
True-positive, *N*	82	560	227	415
True-negative, *N*	416	1,250	550	1,116
False-positive, *N*	6	27	9	24
False-negative, *N*	35	84	42	77
Sensitivity (95% CI)	0.70 (0.62–0.78)	0.87 (0.84–0.90)	0.84 (0.80–0.89)	0.84 (0.81–0.88)
Specificity (95% CI)	0.99 (0.97–1.00)	0.98 (0.97–0.99)	0.98 (0.97–0.99)	0.98 (0.97–0.99)
Kappa score (95% CI)	0.75 (0.68–0.83)	0.87 (0.84–0.89)	0.86 (0.82–0.89)	0.85 (0.82–0.88)

CI, confidence interval.

In the antihypertensive medications group, education years were associated with sensitivity (over 12 years, 0.99; 12 or fewer years; 0.94) and the association was still observed after multivariate adjustment (OR 0.19; 95% CI, 0.05–0.81). In contrast, in the dyslipidemia medications group, sex (males, 0.71; females, 0.92), education years (over 12 years, 0.91; 12 or fewer years; 0.83), and smoking status (current smoker, 0.61; non-current smoker, 0.86) were associated with sensitivity. The associations were still observed after multivariate adjustment for sex (OR 4.15; 95% CI, 2.54–6.77), education years (OR 0.44; 95% CI, 0.23–0.85), and smoking status (OR 2.19; 95% CI, 1.09–4.38) (Table 4).

**Table 4.** Odds ratios (with 95% confidence intervals) for individual factors associated with failure to report regularly dispensed medications

	Antihypertensive medication		Diabetes medication		Dyslipidemia medication	
		
	OR (95% CI)	Adjusted OR (95% CI)	OR (95% CI)	Adjusted OR (95% CI)	OR (95% CI)	Adjusted OR (95% CI)
**Sex**	1.23 (0.69–2.18)	1.67 (0.87–3.22)	2.47 (0.67–9.13)	2.53 (0.57–11.2)	**4.83 (3.17–7.35)**	**4.15 (2.54–6.77)**
**Age**	1.47 (0.72–2.98)	1.38 (0.64–2.99)	1.02 (0.31–3.37)	0.86 (0.22–3.32)	1.08 (0.69–1.70)	1.28 (0.77–2.13)
**Married**	1.32 (0.55–3.16)	1.46 (0.56–3.84)	0.89 (0.19–4.23)	0.60 (0.11–3.24)	1.31 (0.75–2.27)	0.90 (0.50–1.62)
**More than 12 years education**	**0.18 (0.04–0.77)**	**0.19 (0.05–0.81)**	1.75 (0.46–6.68)	1.66 (0.39–7.00)	**0.48 (0.25–0.89)**	**0.44 (0.23–0.85)**
**Currently working**	1.01 (0.57–1.80)	1.19 (0.64–2.21)	0.88 (0.29–2.62)	1.16 (0.33–4.06)	1.32 (0.89–1.96)	0.99 (0.63–1.56)
**Current smoker**	0.83 (0.29–2.36)	1.06 (0.35–3.19)	2.23 (0.58–8.58)	1.87 (0.42–8.45)	**3.79 (2.00–7.20)**	**2.19 (1.09–4.38)**
**Current drinker**	0.51 (0.24–1.11)	**0.40 (0.17–0.95)**	0.95 (0.25–3.53)	0.57 (0.13–2.50)	2.85 (1.80–4.50)	1.28 (0.75–2.19)
**Regular exerciser**	1.26 (0.71–2.26)	1.23 (0.67–2.27)	2.89 (0.97–8.61)	2.50 (0.74–8.44)	1.00 (0.66–1.50)	1.00 (0.64–1.56)

CI, confidence interval; OR, odds ratio.

The following factors were used as controls: female sex, 35–64 years, no marital status, 12 or fewer years of education years, not currently working, non-current smoker, non-current drinker and non-regular exerciser.

Next, we conducted the same subgroup analyses divided by concurrent therapeutic areas (eTable 5). Sex was associated with sensitivity in those with dyslipidemia and those with hypertension and dyslipidemia. Education years were also associated with sensitivity in those with hypertension and dyslipidemia. The associations were still observed after multivariate adjustment (eTable 6). We also conducted the same subgroup analyses divided by sex (eTable 7 and eTable 8).

In the dyslipidemia medications group in male participants, sensitivity was associated with education years (over 12 years, 0.84; 12 or fewer years, 0.68) even after multivariable analysis (OR 0.41; 95% CI, 0.18–0.93) (eTable 9). Further analyses stratifying the same subgroup by education years and smoking status showed the similar tendencies, even after multivariate adjustment. In the group of 12 or fewer years of education years with dyslipidemia medications, sex (males, 0.68; females, 0.91) and smoking status (current smoker, 0.56; non-current smoker, 0.84) were associated with sensitivity (data not shown). Furthermore, sex (males, 0.73; females, 0.92) and education years (over 12 years, 0.92; 12 or fewer years, 0.84) were associated with sensitivity in the group of non-current smoker with dyslipidemia medications (data not shown). These associations were still observed even after multivariate adjustment (eTable 10 and eTable 11).

The characteristics of concordance and discordance groups which affected not only sensitivity but also specificity are shown in eTable 12. The following determinants were associated with discordance: sex (OR 1.69; 95% CI, 1.04–2.74), age (OR 2.06; 95% CI, 1.18–3.59), and education years (OR 0.42; 95% CI, 0.20–0.88) in the antihypertensive medications group; sex (OR 3.62; 95% CI, 1.74–7.51) in the diabetes medications group; and sex (OR 2.33; 95% CI, 1.57–3.46) and age (OR 1.79; 95% CI, 1.19–2.69) in the dyslipidemia medications group (data not shown).

## DISCUSSION

In this study, we found that self-reported medication use had high validity for predicting regular medication use, and that sensitivity for dyslipidemia medication use was lower than those for the other lifestyle-related diseases. Our data provide convincing evidence that self-reported medication use for lifestyle-related diseases is a valid measure to capture regular medication use in a cohort study. Moreover, potential individual determinants, such as sex, education years and smoking status were related with discordance in self-reported medication use for dyslipidemia.

### Medication use information from a self-reported questionnaire and health insurance claims

In this study, we compared the medication use from a self-reported questionnaire with health insurance claims. A previous study showed the sensitivity of information from hospital files, structured interviews and insurance claims data comparing with medication-containing blood samples.^22^ Although the study reported that there were no significant differences between methods, the sensitivity of information from insurance claims data was the highest (0.89 for interview and 0.93 for insurance claims data). According to this result, we considered that insurance claims data would be one of the useful tools to capture the regular medication users from the medication users measured with the self-reported questionnaire in this study.

### 3- and 6-month fixed time windows

No obvious differences in results were observed between 3- and 6-month fixed time windows by sex and concurrent therapeutic areas. Medications for lifestyle-related diseases often need to be taken on a regular basis for a long time, and are often prescribed in quantities for courses of 3 months duration or less. This might have led us to recount the same participants as in the 3-month fixed time window even when we fixed the time window for 6 months.

A previous population-based study in Japan validated self-reported medication use for lifestyle-related disease in 54,712 participants using a 3-month fixed time window for pharmacy health insurance claims.^6^ Their reported sensitivities for antihypertensive medications (92.4%) and dyslipidemia medications (86.2%) were similar to our present results, but their sensitivity for diabetes medications (82.6%) was lower. The reason for this discrepancy is likely due to the type of health insurance claims covered—their validation was done using health insurance claims for pharmacy only, whereas we used claims for both medicine and pharmacy, which provided for more accurate results. Dyslipidemia medication use showed lower sensitivity than the other medication uses in both our present and this previous study.^6^ Awareness level of dyslipidemia is reported to be lower than that of other lifestyle-related diseases such as hypertension.^23^ Self-recognition of health condition is also reported to affect sensitivity.^10^

To our knowledge, our present paper is one of only a few population-based validation studies of self-reported medication use which have covered all of the participants’ health insurance claims.

### Determinants of discordance of self-reported medication use

We found that type of medication, sex, age, education years and smoking status were associated with the accuracy of self-reported medication use. The sensitivity of participants using medications for dyslipidemia was lower than those for the other medications. Males who studied 12 or fewer years and who had a current smoking habit showed lower sensitivity than those who studied more than 12 years and those who were non-current smokers in the dyslipidemia medications group.

Although a number of population-based studies have reported the validity of self-reported medication use, few studies have explored the individual determinants of discordance for self-reported medication use.^7^^,^^9^^,^^10^ A study from Scotland which validated self-reported medication use for cholesterol-lowering medications and antihypertensive medications in 9,043 participants has shown the predictors of discordance that affected sensitivity.^7^ The Scotland study observed that sociodemographic information, including sex, age, marital status, education years, and smoking status did not affect discordance for cholesterol-lowering medication use, but found that female sex, younger age, and smoker were associated with increased discordance for antihypertensive medication use.^7^

The reason only our study identified sex, education years and smoking status as determinants of discordance for dyslipidemia medication use may be due to slight differences among studies in data collection. Whereas our study collected medication data for dyslipidemia medications, the Scottish study collected data on cholesterol-lowering medications only, and might not include medications for hypertriglyceridemia or hypo HDL-cholesterolemia.^7^

Studies from Finland and Ireland have explored the predictors of discordance which affected not only sensitivity but also specificity. In the Finnish study, the diabetes medication use in 7,625 participants was validated and the study has reported that none of the sociodemographic information was associated with the discordance.^9^ The Irish study validated calcium channel blockers, diabetes medication and lipid-modifying agent use in 2,621 participants and it has reported that older age was associated with increased discordance for the use of calcium channel blockers,^10^ which showed the same tendency as the antihypertensive medications group in the Tsuruoka Metabolomics Cohort Study. There is a possibility that the predictors of discordance might be different depending on the definition of discordance.

Although previous studies did not identify education status as a determinant of discordance for self-reported medication use for lifestyle-related disease, a few studies of antidepressant use reported that a lack of high education was associated with worse recall.^4^^,^^7^^,^^9^ We assume that participants without high education might take the medications not knowing their efficacy, due to either a lack of knowledge, lack of interest in the treatment, or poor health awareness, such as smoking cigarettes, particularly with regard to diseases with few or no symptoms, such as dyslipidemia.

### Study strengths and weaknesses

Among its strengths, this study was conducted by linkage of population-based cohort data with both medical and pharmacy health insurance claims. Our use of information on prescribed medications dispensed from hospitals and pharmacies enabled us to draw accurate results. Furthermore, our detailed analyses by the factors that would affect sensitivity strongly supported the associations, especially in those with dyslipidemia medications.

Several limitations of our study also warrant mention. First, we covered only a part of participants in this study, namely beneficiaries of NHI and Medical Care System for the Advanced Elderly. The selection of participants might lead to the older age demographic in this study. Further study will be required for beneficiaries of Employees’ Health Insurance, which include most participants aged younger than 65 years. Second, the health insurance claims data may be insufficient for participants who newly changed their coverage from Employees’ Health Insurance to NHI. This might have increased the number of false-positive results. Third, adherence to medication was not considered. Although we observed high sensitivity and specificity for each medication, we do not know if the participants took the medications correctly as indicated, because the prescription records provide only the fact that patients have received the medications. In this study, we could observe the proportion of those with medications, but there is a possibility that some of the participants with low adherence are included in regular medication users. Fourth, the generalizability of this study to other questionnaires might be limited as we analyzed the participants who joined the cohort study. The participants who joined a cohort study might report their medication use more accurately than those who did not. Fifth, we also conducted the validation by concurrent therapeutic areas; however, further study will be needed by increasing the number of participants. Sixth, in the analyses by the factors which would affect sensitivity, the associations were not determined enough by the response variables due to a small number of failures, especially in those with hypertension and diabetes. Further study also will be needed by increasing the number of participants in regard to this point. Finally, only medications for lifestyle-related diseases were validated. Further study will be needed with other medications.

In conclusion, we found that the self-reported medication use for lifestyle-related diseases was a valid measure to capture regular medication use in a cohort study. Sensitivity for dyslipidemia medications was lower than those for the others. Dyslipidemia medication, sex, number of years of education, and smoking habit were associated with discordance which affected sensitivity in self-reporting.

## APPENDIX A. SUPPLEMENTARY DATA

eTable 1. Medications for hypertension

eTable 2. Medications for diabetes

eTable 3. Medications for dyslipidemia

eTable 4. Validity of self-reported medication use by concurrent therapeutic areas

eTable 5. Validity of self-reported medication use among subgroups with a 3-month fixed time window by concurrent therapeutic areas

eTable 6. Odds ratios (with 95% confidence intervals) for individual factors associated with failure to report regularly dispensed medications by concurrent therapeutic areas

eTable 7. Validity of self-reported medication use among subgroups with a 3-month fixed time window in males

eTable 8. Validity of self-reported medication-use among subgroups with a 3-month fixed time window in females

eTable 9. Odds ratios (with 95% confidence intervals) for individual determinants associated with failure to report regularly dispensed medications by sex

eTable 10. Odds ratios for individual determinants associated with failure to report regularly dispensed medications by education years

eTable 11. Odds ratios for individual determinants associated with failure to report regularly dispensed medications by smoking status

eTable 12. Characteristics of the study population by concordance and discordance groups between health insurance claims and self-reported medication use

eMaterials 1. The standardized self-administrated questionnaire which was used in the Tsuruoka Metabolomics Cohort Study
