# Erratum to “Validity Assessment of Self-reported Medication Use for Hypertension, Diabetes, and Dyslipidemia in a Pharmacoepidemiologic Study by Comparison With Health Insurance Claims”

**DOI:** 10.2188/jea.JE20210109

**Published:** 2021-09-05

**Authors:** Minako Matsumoto, Sei Harada, Miho Iida, Suzuka Kato, Mizuki Sata, Aya Hirata, Kazuyo Kuwabara, Ayano Takeuchi, Daisuke Sugiyama, Tomonori Okamura, Toru Takebayashi

**Affiliations:** Department of Preventive Medicine and Public Health, School of Medicine, Keio University, Tokyo, Japan

In the original publication of the article,^1^ some of the generic names and their Anatomical Therapeutic Chemical (ATC) codes were incorrectly stated in eTable 1, eTable 2, and eTable 3. Some of the generic names and their ATC codes have been added or deleted. Additionally, some of the generic names have been amended to the official names. The correct tables are presented with this erratum.

In eTable 1, the following generic names and their ATC codes have been added: Azilsartan · amlodipine besilate, Telmisartan · amlodipine besilate, Irbesartan · amlodipine besilate, Aliskiren fumarate, Eplerenone, Prazosin hydrochloride, Bunazosin hydrochloride, Terazosin hydrochloride hydrate, Urapidil, Doxazosin mesilate, Clonidine hydrochloride, Methyldopa hydrate, Guanabenz acetat, Reserpine, Hydralazine hydrochloride, Benzylhydrochlorothiazide · reserpine combinations, Ambrisentan, Selexipag, Tadalafil, Sildenafil citrate, Macitentan, Riociguat, Bosentan hydrate, Beraprost sodium, Nifedipine, Nicardipine hydrochloride, Nilvadipine, Manidipine hydrochloride, Barnidipine hydrochloride, Efonidipine hydrochloride ethanolate, Felodipine, Cilnidipine, Aranidipine, Verapamil hydrochloride, Azelnidipine, Amlodipine besilate, Nisoldipine, Nitrendipine, Diltiazem hydrochloride, Benidipine hydrochloride, and Atorvastatin calcium hydrate · amlodipine besilate. Additionally, Mozavaptane and Tolvaptan and their ATC codes have been deleted.

Furthermore, the names listed as Propranolol, Bufetolol, Carteolol, Metoprolol, Betaxolol, Acebutolol, Celiprolol, Labetalol, Bevantolol, Amosulalol, Arotinolol, Enalapril, Delapril, Cilazapril, Lisinopril, Benazepril, Imidapril, Temocapril, Quinapril, Perindopril, Candesartan, Losartan, Azilsartan medoxomil, Losartan and diuretics, Candesartan and diuretics, Valsartan and diuretics, Telmisartan and diuretics, Irbesartan and diuretics, Olmesartan medoxomil and azelnidipine, Valsartan and cilnidipine, Valsartan and amlodipine, Candesartan and amlodipine, and Telmisartan and amlodipine and Hydrochlorothiazide have also been amended to Propranolol hydrochloride, Bufetolol hydrochloride, Carteolol hydrochloride, Metoprolol tartrate, Betaxolol hydrochloride, Acebutolol hydrochloride, Celiprolol hydrochloride, Labetalol hydrochloride, Bevantolol hydrochloride, Amosulalol hydrochloride, Arotinolol hydrochloride, Enalapril maleate, Delapril hydrochloride, Cilazapril hydrate, Lisinopril hydrate, Benazepril hydrochloride, Imidapril hydrochloride, Temocapril hydrochloride, Quinapril hydrochloride, Perindopril erbumine, Candesartan cilexetil, Losartan potassium, Azilsartan, Losartan potassium · hydrochlorothiazide, Candesartan cilexetil · hydrochlorothiazide, Valsartan · hydrochlorothiazide, Telmisartan · hydrochlorothiazide, Irbesartan · trichlormethiazide, Olmesartan medoxomil · azelnidipine, Valsartan · cilnidipine, Valsartan · amlodipine besilate, Candesartan cilexetil · amlodipine besilate, and Telmisartan · amlodipine besilate · hydrochlorothiazide, respectively.

In eTable 2, Tolbutamide and its ATC code have been added to the list. Additionally, the correct ATC codes have been assigned to the following medications: Glyclopyramide, Luseogliflozin hydrate, Teneligliptin and canagliflozin, and Sitagliptin and ipragliflozin.

The names listed as buformin, metformin, Pioglitazone, Mitiglinide, Sitagliptin, Alogliptin, Teneligliptin, Saxagliptin, Trelagliptin, Ipragliflozin, Tofogliflozin, Dapagliflozin, Canagliflozin, Metformin and pioglitazone, Metformin and vildagliptin, Glimepiride and pioglitazone, Mitiglinide calcium hydrate and voglibose, Pioglitazone and alogliptin, Metformin and alogliptin, Teneligliptin and canagliflozin, and Sitagliptin and ipragliflozin have also been amended to Buformin hydrochloride, Metformin hydrochloride, Pioglitazone hydrochloride, Mitiglinide calcium hydrate, Sitagliptin phosphate hydrate, Alogliptin benzoate, Teneligliptin hydrobromide hydrate, Saxagliptin hydrate, Trelagliptin succinate, Ipragliflozin L-proline, Tofogliflozin hydrate, Dapagliflozin propylene glycolate hydrate, Canagliflozin hydrate, Metformin hydrochloride · pioglitazone hydrochloride, Metformin hydrochloride · vildagliptin, Glimepiride · pioglitazone hydrochloride, Mitiglinide calcium hydrate · voglibose, Pioglitazone hydrochloride · alogliptin benzoate, Metformin hydrochloride · alogliptin benzoate, Teneligliptin hydrobromide hydrate · canagliflozin hydrate, and Sitagliptin phosphate hydrate · ipragliflozin L-proline, respectively.

In eTable 3, Bezafibrate and Elastase ES and their ATC codes have been added. The names listed as Pravastatin, Fluvastatin, Atorvastatin, Pitavastatin, Rosuvastatin, Atorvastatin and amlodipine, Atorvastatin and ezetimibe, Lomitapide, Colestilan, Gamma Oryzanol, Dextran, and Ethyl Icosapentate have also been amended to Pravastatin sodium, Fluvastatin sodium, Atorvastatin calcium hydrate, Pitavastatin calcium hydrate, Rosuvastatin calcium, Atorvastatin calcium hydrate · amlodipine besilate, Atorvastatin calcium hydrate · ezetimibe, Lomitapide mesilate, Colestimide, Gamma oryzanol, Dextran sulfate sodium sulfur 18, and Ethyl icosapentate, respectively.

These errors do not affect the results or the implications of the study findings. The authors apologize for these errors.

## APPENDIX A. SUPPLEMENTARY DATA

eTable 1. Medications for hypertension

eTable 2. Medications for diabetes

eTable 3. Medications for dyslipidemia
