# Supplementary material for: Validity Assessment of Self-reported Medication Use for Hypertension, Diabetes, and Dyslipidemia in a Pharmacoepidemiologic Study by Comparison With Health Insurance Claims
Source: J Epidemiol. 2021 Sep 5;31(9):495–502. doi: 10.2188/jea.JE20200089 (PMC8328856; doi:10.2188/jea.JE20200089)
Supplement: Supplementary file 1 [file je-31-495-s001.pdf]

eTable 1. Medications for hypertension

| Therapeutic category of drugs in Japan | | Generic name | ATC codes | |
|---|---|---|---|---|
| 21: Cardiovascular agents | 212: Antiarrhythmic agents | Propranolol | C07AA05 | |
| | | Bufetolol | C07 | |
| | | Nadolol | C07AA12 | |
| | | Pindolol | C07AA03 | |
| | | Carteolol | C07AA15 | S01ED05 |
| | | Metoprolol | C07AB02 | |
| | | Atenolol | C07AB03 | |
| | | Bisoprolol | C07AB07 | |
| | | Betaxolol | C07AB05 | S01ED02 |
| | | Acebutolol | C07AB04 | |
| | | Celiprolol | C07AB08 | |
| | | Labetalol | C07AG01 | |
| | | Carvedilol | C07AG02 | |
| | | Bevantolol | C07AB06 | |
| | | Amosulalol | C07 | |
| | | Arotinolol | C07 | |
| | | Nipradilol | C07 | |
| | 213: Diuretics | Trichlormethiazide | C03AA06 | |
| | | Hydrochlorothiazide | C03AA03 | |
| | | Benzylhydrochlorothiazide | C03 | |
| | | Indapamide | C03BA11 | |
| | | Tripamide | C03 | |
| | | Meticrane | C03BA09 | |
| | | Mefruside | C03BA05 | |
| | | Furosemide | C03CA01 | |
| | | Bumetanide | C03CA02 | |
| | | Azosemide | C03CA | |
| | | Torasemide | C03CA04 | |
| | | Spironolactone | C03DA01 | |
| | | Triamterene | C03DB02 | |
| | | Mozavaptane | C03 | |
| | | Tolvaptan | C03XA01 | |

ATC, Anatomical Therapeutic Chemical.

**eTable 1.** Medications for hypertension (continued)

| Therapeutic category of drugs in Japan | | Generic name | ATC codes |
|---|---|---|---|
| 21: Cardiovascular agents | 214: Hypertensions | Captopril | C09AA01 |
| | | Enalapril | C09AA02 |
| | | Alacepril | C09AA |
| | | Delapril | C09AA12 |
| | | Cilazapril | C09AA08 |
| | | Lisinopril | C09AA03 |
| | | Benazepril | C09AA07 |
| | | Imidapril | C09AA16 |
| | | Temocapril | C09AA14 |
| | | Quinapril | C09AA06 |
| | | Trandolapril | C09AA10 |
| | | Perindopril | C09AA04 |
| | | Candesartan | C09CA06 |
| | | Losartan | C09CA01 |
| | | Valsartan | C09CA03 |
| | | Telmisartan | C09CA07 |
| | | Olmesartan medoxomil | C09CA08 |
| | | Irbesartan | C09CA04 |
| | | Azilsartan medoxomil | C09CA09 |
| | | Losartan and diuretics | C09DA01 |
| | | Candesartan and diuretics | C09DA06 |
| | | Valsartan and diuretics | C09DA03 |
| | | Telmisartan and diuretics | C09DA07 |
| | | Irbesartan and diuretics | C09DA04 |
| | | Olmesartan medoxomil and azelnidipine | C09DB |
| | | Valsartan and cilnidipine | C09DB |
| | | Valsartan and amlodipine | C09DB01 |
| | | Candesartan and amlodipine | C09DB07 |
| | | Telmisartan and amlodipine and Hydrochlorothiazide | C09DX |

ATC, Anatomical Therapeutic Chemical.

**eTable 2.** Medications for diabetes

| Therapeutic category of drugs in Japan | | Generic name | ATC codes |
|---|---|---|---|
| 39: Other agents affecting metabolism | 396: Antidiabetic agents | Chlorpropamide | A10BB02 |
| | | Acetohexamide | A10BB31 |
| | | Glyclopyramide | A10BC |
| | | Glibenclamide | A10BB01 |
| | | Gliclazide | A10BB09 |
| | | Glimepiride | A10BB12 |
| | | buformin | A10BA03 |
| | | metformin | A10BA02 |
| | | Pioglitazone | A10BG03 |
| | | Acarbose | A10BF01 |
| | | Voglibose | A10BF03 |
| | | Miglitol | A10BF02 |
| | | Nateglinide | A10BX03 |
| | | Mitiglinide | A10BX08 |
| | | Repaglinide | A10BX02 |
| | | Sitagliptin | A10BH01 |
| | | Vildagliptin | A10BH02 |
| | | Alogliptin | A10BH04 |
| | | Linagliptin | A10BH05 |
| | | Teneligliptin | A10BH |
| | | Anagliptin | A10BH |
| | | Saxagliptin | A10BH03 |
| | | Trelagliptin | A10BH |
| | | Omarigliptin | A10BH |
| | | Ipragliflozin | A10BK05 |
| | | Tofogliflozin | A10BK |
| | | Dapagliflozin | A10BK01 |
| | | Luseogliflozin hydrate | A10BK |
| | | Canagliflozin | A10BK02 |
| | | Empagliflozin | A10BK03 |
| | | Metformin and pioglitazone | A10BD05 |

ATC, Anatomical Therapeutic Chemical.

**eTable 2.** Medications for diabetes (continued)

| Therapeutic category of drugs in Japan | Generic name | ATC codes |
|---|---|---|
| | Metformin and vildagliptin | A10BD08 |
| | Glimepiride and pioglitazone | A10BD06 |
| | Mitiglinide calcium hydrate and voglibose | A10BD |
| | Pioglitazone and alogliptin | A10BD09 |
| | Metformin and alogliptin | A10BD13 |
| | Teneligliptin and canagliflozin | A10BH |
| | Sitagliptin and ipragliflozin | A10BH |

ATC, Anatomical Therapeutic Chemical.

**eTable 3.** Medications for dyslipidemia

| Therapeutic category of drugs in Japan | | Generic name | ATC codes | |
|---|---|---|---|---|
| 21: Cardiovascular agents | 218: Hyperlipidemia agents | Pravastatin | C10AA03 | |
| | | Simvastatin | C10AA01 | |
| | | Fluvastatin | C10AA04 | |
| | | Atorvastatin | C10AA05 | |
| | | Pitavastatin | C10AA08 | |
| | | Rosuvastatin | C10AA07 | |
| | | Atorvastatin and amlodipine | C10BX03 | |
| | | Clofibrate | C10AB01 | |
| | | Clinofibrate | C10 | |
| | | Clinofibrate | C10AB02 | |
| | | Fenofibrate | C10AB05 | |
| | | Pemafibrate | C10 | |
| | | Ezetimibe | C10AX09 | |
| | | Atorvastatin and ezetimibe | C10BA05 | |
| | | Lomitapide | C10AX12 | |
| | | Nicomol | C10 | |
| | | Niceritrol | C10AD01 | |
| | | Colestyramine | C10AC01 | |
| | | Colestilan | C10AC | V03AE06 |
| | | Probucol | C10AX02 | |
| | | Gamma Oryzanol | C10 | |
| | | Dextran | C10 | B05AA05 |
| | | Polyenephosphatidylcholine | C10 | |
| | | Ethyl Icosapentate | B01AC | |
| | | Omega-three-acid ethyl esters | C10AX | |

ATC, Anatomical Therapeutic Chemical.

**eTable 4.** Validity of self-reported medication use by concurrent therapeutic areas

| | Antihypertensive medication | DM medication | DL medication | Antihypertensive + DM medications | Antihypertensive + DL medications | DM + DL medications | Antihypertensive + DM + DL medications |
|---|---|---|---|---|---|---|---|
| **Three-month fixed time window** | | | | | | | |
| True-positive, N | 447 | 33 | 253 | 48 | 263 | 31 | 61 |
| True-negative, N | 1922 | 2410 | 2158 | 2384 | 2103 | 2419 | 2378 |
| False-positive, N | 80 | 26 | 25 | 24 | 21 | 9 | 13 |
| False-negative, N | 23 | 3 | 36 | 16 | 85 | 13 | 20 |
| Sensitivity (95% CI) | 0.95 (0.93-0.97) | 0.92 (0.83-1.00) | 0.88 (0.84-0.91) | 0.75 (0.64-0.86) | 0.76 (0.71-0.80) | 0.70 (0.57-0.84) | 0.75 (0.66-0.85) |
| Specificity (95% CI) | 0.96 (0.95-0.97) | 0.99 (0.99-0.99) | 0.99 (0.98-0.99) | 0.99 (0.99-0.99) | 0.99 (0.99-0.99) | 1.00 (0.99-1.00) | 0.99 (0.99-1.00) |
| Kappa score (95% CI) | 0.87 (0.85-0.90) | 0.69 (0.58-0.80) | 0.88 (0.85-0.91) | 0.70 (0.61-0.79) | 0.81 (0.77-0.84) | 0.73 (0.63-0.84) | 0.78 (0.71-0.85) |
| **Six-month fixed time window** | | | | | | | |
| True-positive, N | 447 | 33 | 260 | 49 | 264 | 31 | 61 |
| True-negative, N | 1919 | 2409 | 2160 | 2385 | 2098 | 2417 | 2375 |
| False-positive, N | 80 | 26 | 18 | 23 | 20 | 9 | 13 |
| False-negative, N | 26 | 4 | 34 | 15 | 90 | 15 | 23 |
| Sensitivity (95% CI) | 0.95 (0.92-0.97) | 0.89 (0.79-0.99) | 0.88 (0.85-0.92) | 0.77 (0.66-0.87) | 0.75 (0.70-0.79) | 0.67 (0.54-0.81) | 0.73 (0.63-0.82) |
| Specificity (95% CI) | 0.96 (0.95-0.97) | 0.99 (0.99-0.99) | 0.99 (0.99-1.00) | 0.99 (0.99-0.99) | 0.99 (0.99-0.99) | 1.00 (0.99-1.00) | 0.99 (0.99-1.00) |
| Kappa score (95% CI) | 0.87 (0.84-0.89) | 0.68 (0.57-0.79) | 0.90 (0.87-0.92) | 0.71 (0.63-0.80) | 0.80 (0.77-0.84) | 0.72 (0.61-0.82) | 0.76 (0.69-0.84) |

CI, confidence interval; DL, Dyslipidemia medication; DM, Diabetes medication.

**eTable 5.** Validity of self-reported medication use among subgroups with a 3-month fixed time window by concurrent therapeutic areas

| | Sex | | Age | | Married | |
|---|---|---|---|---|---|---|
| | Males | Females | 65-78 years | 35-64 years | Yes | No |
| **Antihypertensive medication** | | | | | | |
| True-positive, N | 251 | 196 | 309 | 138 | 382 | 63 |
| True-negative, N | 813 | 1109 | 1129 | 793 | 1584 | 325 |
| False-positive, N | 52 | 28 | 64 | 16 | 70 | 9 |
| False-negative, N | 12 | 11 | 18 | 5 | 21 | 2 |
| Sensitivity (95% CI) | 0.95 (0.93-0.98) | 0.95 (0.92-0.98) | 0.94 (0.92-0.97) | 0.97 (0.93-1.00) | 0.95 (0.93-0.97) | 0.97 (0.93-1.00) |
| Specificity (95% CI) | 0.94 (0.92-0.96) | 0.98 (0.97-0.98) | 0.95 (0.93-0.96) | 0.98 (0.97-0.99) | 0.96 (0.95-0.97) | 0.97 (0.96-0.99) |
| Kappa score (95% CI) | 0.85 (0.81-0.88) | 0.89 (0.86-0.93) | 0.85 (0.82-0.88) | 0.92 (0.88-0.95) | 0.87 (0.84-0.89) | 0.90 (0.85-0.96) |
| **Dyslipidemia medication** | | | | | | |
| True-positive, N | 51 | 202 | 168 | 85 | 201 | 50 |
| True-negative, N | 1048 | 1110 | 1305 | 853 | 1807 | 338 |
| False-positive, N | 10 | 15 | 17 | 8 | 20 | 5 |
| False-negative, N | 19 | 17 | 30 | 6 | 29 | 6 |
| Sensitivity (95% CI) | 0.73 (0.62-0.83) | 0.92 (0.89-0.96) | 0.85 (0.80-0.90) | 0.93 (0.88-0.99) | 0.87 (0.83-0.92) | 0.89 (0.81-0.97) |
| Specificity (95% CI) | 0.99 (0.98-1.00) | 0.99 (0.98-0.99) | 0.99 (0.98-0.99) | 0.99 (0.98-1.00) | 0.99 (0.98-0.99) | 0.99 (0.97-1.00) |
| Kappa score (95% CI) | 0.77 (0.68-0.88) | 0.91 (0.88-0.94) | 0.86 (0.82-0.90) | 0.92 (0.87-0.96) | 0.88 (0.84-0.91) | 0.88 (0.82-0.95) |
| **Antihypertensive + Dyslipidemia medications** | | | | | | |
| True-positive, N | 85 | 178 | 206 | 57 | 213 | 50 |
| True-negative, N | 979 | 1124 | 1226 | 877 | 1756 | 332 |
| False-positive, N | 8 | 13 | 19 | 2 | 16 | 5 |
| False-negative, N | 56 | 29 | 69 | 16 | 72 | 12 |
| Sensitivity (95% CI) | 0.60 (0.52-0.68) | 0.86 (0.81-0.91) | 0.75 (0.70-0.80) | 0.78 (0.69-0.88) | 0.75 (0.70-0.80) | 0.81 (0.71-0.90) |
| Specificity (95% CI) | 0.99 (0.99-1.00) | 0.99 (0.98-0.99) | 0.98 (0.98-0.99) | 1.00 (0.99-1.00) | 0.99 (0.99-1.00) | 0.99 (0.97-1.00) |
| Kappa score (95% CI) | 0.70 (0.63-0.77) | 0.88 (0.84-0.91) | 0.64 (0.47-0.82) | 0.85 (0.79-0.92) | 0.80 (0.77-0.84) | 0.83 (0.75-0.91) |

CI, confidence interval.

**eTable 5.** Validity of self-reported medication use among subgroups with a 3-month fixed time window by concurrent therapeutic areas (continued)

| | More than 12 years education | | Currently working | | Current smoker | |
|---|---|---|---|---|---|---|
| | Yes | No | Yes | No | Yes | No |
| **Antihypertensive medication** | | | | | | |
| True-positive, N | 86 | 359 | 259 | 187 | 54 | 389 |
| True-negative, N | 414 | 1,498 | 1,187 | 724 | 241 | 1,673 |
| False-positive, N | 8 | 71 | 44 | 36 | 8 | 72 |
| False-negative, N | 1 | 22 | 12 | 11 | 2 | 21 |
| Sensitivity (95% CI) | 0.99 (0.97–1.00) | 0.94 (0.92–0.97) | 0.96 (0.93–0.98) | 0.94 (0.91–0.98) | 0.96 (0.92–1.00) | 0.95 (0.93–0.97) |
| Specificity (95% CI) | 0.98 (0.97–1.00) | 0.95 (0.94–0.97) | 0.96 (0.95–0.97) | 0.95 (0.94–0.97) | 0.97 (0.95–0.99) | 0.96 (0.95–0.97) |
| Kappa score (95% CI) | 0.94 (0.90–0.98) | 0.86 (0.83–0.88) | 0.88 (0.85–0.91) | 0.86 (0.82–0.90) | 0.90 (0.83–0.96) | 0.87 (0.84–0.89) |
| **Dyslipidemia medication** | | | | | | |
| True-positive, N | 53 | 199 | 131 | 121 | 9 | 244 |
| True-negative, N | 446 | 1,701 | 1,342 | 806 | 290 | 1,856 |
| False-positive, N | 6 | 19 | 12 | 13 | 1 | 24 |
| False-negative, N | 4 | 31 | 17 | 18 | 5 | 31 |
| Sensitivity (95% CI) | 0.93 (0.86–1.00) | 0.87 (0.82–0.91) | 0.89 (0.83–0.94) | 0.87 (0.81–0.93) | 0.64 (0.39–0.89) | 0.89 (0.85–0.92) |
| Specificity (95% CI) | 0.99 (0.98–1.00) | 0.99 (0.98–0.99) | 0.99 (0.99–1.00) | 0.98 (0.98–0.99) | 1.00 (0.99–1.00) | 0.99 (0.98–0.99) |
| Kappa score (95% CI) | 0.90 (0.84–0.96) | 0.87 (0.84–0.91) | 0.89 (0.85–0.93) | 0.87 (0.82–0.91) | 0.74 (0.54–0.94) | 0.88 (0.85–0.91) |
| **Antihypertensive + Dyslipidemia medications** | | | | | | |
| True-positive, N | 53 | 210 | 131 | 132 | 12 | 249 |
| True-negative, N | 443 | 1,647 | 1,319 | 772 | 282 | 1,811 |
| False-positive, N | 5 | 16 | 8 | 13 | 2 | 19 |
| False-negative, N | 8 | 77 | 44 | 41 | 9 | 76 |
| Sensitivity (95% CI) | 0.87 (0.78–0.95) | 0.73 (0.68–0.78) | 0.75 (0.68–0.81) | 0.76 (0.70–0.83) | 0.57 (0.36–0.78) | 0.77 (0.72–0.81) |
| Specificity (95% CI) | 0.99 (0.98–1.00) | 0.99 (0.99–1.00) | 0.99 (0.99–1.00) | 0.98 (0.97–0.99) | 0.99 (0.98–1.00) | 0.99 (0.98–0.99) |
| Kappa score (95% CI) | 0.88 (0.81–0.94) | 0.79 (0.75–0.83) | 0.82 (0.77–0.86) | 0.80 (0.74–0.85) | 0.67 (0.48–0.85) | 0.81 (0.78–0.85) |

CI, confidence interval.

**eTable 5.** Validity of self-reported medication use among subgroups with a 3-month fixed time window by concurrent therapeutic areas (continued)

| | Current drinker | | Regular exerciser | |
|---|---|---|---|---|
| | Yes | No | Yes | No |
| **Antihypertensive medication** | | | | |
| True-positive, N | 146 | 300 | 161 | 285 |
| True-negative, N | 367 | 1,547 | 636 | 1,278 |
| False-positive, N | 23 | 54 | 24 | 53 |
| False-negative, N | 3 | 20 | 7 | 16 |
| Sensitivity (95% CI) | 0.98 (0.96–1.00) | 0.94 (0.91–0.96) | 0.96 (0.93–0.99) | 0.95 (0.92–0.97) |
| Specificity (95% CI) | 0.94 (0.92–0.96) | 0.97 (0.96–0.98) | 0.96 (0.95–0.98) | 0.96 (0.95–0.97) |
| Kappa score (95% CI) | 0.88 (0.84–0.93) | 0.87 (0.84–0.90) | 0.89 (0.85–0.93) | 0.87 (0.84–0.90) |
| **Dyslipidemia medication** | | | | |
| True-positive, N | 24 | 229 | 88 | 165 |
| True-negative, N | 508 | 1,638 | 709 | 1,437 |
| False-positive, N | 4 | 21 | 12 | 13 |
| False-negative, N | 3 | 33 | 19 | 17 |
| Sensitivity (95% CI) | 0.89 (0.77–1.00) | 0.87 (0.83–0.91) | 0.82 (0.75–0.89) | 0.91 (0.86–0.95) |
| Specificity (95% CI) | 0.99 (0.98–1.00) | 0.99 (0.98–0.99) | 0.98 (0.97–0.99) | 0.99 (0.99–1.00) |
| Kappa score (95% CI) | 0.87 (0.77–0.96) | 0.88 (0.85–0.91) | 0.83 (0.77–0.89) | 0.91 (0.87–0.94) |
| **Antihypertensive + Dyslipidemia medications** | | | | |
| True-positive, N | 41 | 222 | 95 | 168 |
| True-negative, N | 469 | 1,622 | 687 | 1,404 |
| False-positive, N | 1 | 20 | 12 | 9 |
| False-negative, N | 28 | 57 | 34 | 51 |
| Sensitivity (95% CI) | 0.59 (0.48–0.71) | 0.80 (0.75–0.84) | 0.74 (0.66–0.81) | 0.77 (0.71–0.82) |
| Specificity (95% CI) | 1.00 (0.99–1.00) | 0.99 (0.98–0.99) | 0.98 (0.97–0.99) | 0.99 (0.99–1.00) |
| Kappa score (95% CI) | 0.71 (0.61–0.81) | 0.83 (0.79–0.87) | 0.71 (0.61–0.81) | 0.83 (0.79–0.87) |

CI, confidence interval.

**eTable 6.** Odds ratios (with 95% confidence intervals) for individual factors associated with failure to report regularly dispensed medications by concurrent therapeutic areas

| | Antihypertensive medication | | Dyslipidemia medication | | Antihypertensive + Dyslipidemia medications | |
|---|---|---|---|---|---|---|
| | OR (95% CI) | Adjusted OR [a] (95% CI) | OR (95% CI) | Adjusted OR [a] (95% CI) | OR (95% CI) | Adjusted OR (95% CI) |
| **Sex** | 0.85 (0.37–1.97) | 1.45 (0.56–3.75) | **4.43 (2.15–9.12)** | **6.19 (2.56–15.0)** | **4.04 (2.41–6.79)** | **3.60 (1.95–6.62)** |
| **Age** | 1.61 (0.59–4.42) | 1.35 (0.47–3.90) | **2.53 (1.01–6.31)** | 2.37 (0.81–6.95) | 1.19 (0.64–2.21) | 1.42 (0.71–2.86) |
| **Married** | 1.73 (0.40–7.57) | 1.56 (0.35–7.08) | 1.20 (0.47–3.05) | 0.93 (0.34–2.56) | 1.41 (0.71–2.79) | 1.10 (0.53–2.29) |
| **More than 12 years education** | 0.19 (0.03–1.43) | 0.19 (0.03–1.43) | 0.48 (0.16–1.43) | 0.52 (0.16–1.64) | **0.41 (0.19–0.91)** | **0.36 (0.16–0.83)** |
| **Currently working** | 0.79 (0.34–1.82) | 0.94 (0.39–2.29) | 0.87 (0.43–1.77) | 0.92 (0.40–2.12) | 1.08 (0.66–1.76) | 0.78 (0.44–1.37) |
| **Current smoker** | 0.69 (0.16–3.01) | 0.89 (0.19–4.25) | **4.37 (1.38–13.9)** | 3.19 (0.77–13.2) | **2.46 (1.00–6.05)** | 1.65 (0.61–4.51) |
| **Current drinker** | 0.31 (0.09–1.05) | **0.25 (0.05–0.95)** | 0.87 (0.25–3.04) | 0.27 (0.06–1.13) | **2.66 (1.52–4.67)** | 1.46 (0.73–2.91) |
| **Regular exerciser** | 0.78 (0.31–1.92) | 0.73 (0.29–1.86) | **2.10 (1.04–4.24)** | 1.89 (0.86–4.14) | 1.18 (0.71–1.95) | 1.11 (0.64–1.94) |

CI, confidence interval; OR, odds ratio.

The following factors were used as controls: female sex, 35–64 years, no marital status, 12 or fewer years of education years, not currently working, non-current smoker, non-current drinker and non-regular exerciser.

**eTable 7.** Validity of self-reported medication use among subgroups with a 3-month fixed time window in males

| | Age | | Married | | More than 12 years of education | |
|---|---|---|---|---|---|---|
| | 65–78 years | 35–64 years | Yes | No | Yes | No |
| **Antihypertensive medication** | | | | | | |
| True-positive, N | 322 | 143 | 418 | 43 | 89 | 374 |
| True-negative, N | 315 | 300 | 538 | 73 | 146 | 465 |
| False-positive, N | 15 | 5 | 17 | 3 | 2 | 18 |
| False-negative, N | 21 | 7 | 25 | 2 | 0 | 28 |
| Sensitivity (95% CI) | 0.94 (0.91–0.96) | 0.95 (0.92–0.99) | 0.94 (0.92–0.97) | 0.96 (0.90–1.02) | 1.00 (–) | 0.93 (0.91–0.96) |
| Specificity (95% CI) | 0.95 (0.93–0.98) | 0.98 (0.97–1.00) | 0.97 (0.96–0.98) | 0.96 (0.92–1.00) | 0.99 (0.97–1.01) | 0.96 (0.95–0.98) |
| Kappa score (95% CI) | 0.89 (0.86–0.93) | 0.94 (0.91–0.97) | 0.91 (0.89–0.94) | 0.91 (0.84–0.99) | 0.98 (0.96–1.00) | 0.89 (0.87–0.92) |
| **Diabetes medication** | | | | | | |
| True-positive, N | 87 | 39 | 114 | 10 | 18 | 106 |
| True-negative, N | 561 | 407 | 855 | 107 | 213 | 751 |
| False-positive, N | 17 | 6 | 20 | 3 | 3 | 20 |
| False-negative, N | 8 | 3 | 9 | 1 | 3 | 8 |
| Sensitivity (95% CI) | 0.92 (0.86–0.97) | 0.93 (0.85–1.01) | 0.93 (0.88–0.97) | 0.91 (0.74–1.08) | 0.86 (0.71–1.01) | 0.93 (0.88–0.98) |
| Specificity (95% CI) | 0.97 (0.96–0.98) | 0.99 (0.97–1.00) | 0.98 (0.97–0.99) | 0.97 (0.94–1.00) | 0.99 (0.97–1.00) | 0.97 (0.96–0.99) |
| Kappa score (95% CI) | 0.85 (0.80–0.91) | 0.89 (0.81–0.96) | 0.87 (0.82–0.92) | 0.82 (0.64–0.99) | 0.84 (0.72–0.97) | 0.87 (0.82–0.91) |
| **Dyslipidemia medication** | | | | | | |
| True-positive, N | 133 | 64 | 176 | 19 | 41 | 155 |
| True-negative, N | 469 | 367 | 737 | 93 | 181 | 650 |
| False-positive, N | 9 | 5 | 12 | 2 | 7 | 7 |
| False-negative, N | 62 | 19 | 73 | 7 | 8 | 73 |
| Sensitivity (95% CI) | 0.68 (0.62–0.75) | 0.77 (0.68–0.86) | 0.71 (0.65–0.76) | 0.73 (0.56–0.90) | 0.84 (0.73–0.94) | 0.68 (0.62–0.74) |
| Specificity (95% CI) | 0.98 (0.97–0.99) | 0.99 (0.97–1.00) | 0.98 (0.97–0.99) | 0.98 (0.95–1.01) | 0.96 (0.94–0.99) | 0.99 (0.98–1.00) |
| Kappa score (95% CI) | 0.72 (0.66–0.78) | 0.81 (0.74–0.88) | 0.75 (0.70–0.80) | 0.76 (0.62–0.91) | 0.81 (0.71–0.90) | 0.74 (0.69–0.79) |

CI, confidence interval.

**eTable 7.** Validity of self-reported medication use among subgroups with a 3-month fixed time window in males (continued)

| | Currently working | | Current smoker | | Current drinker | |
|---|---|---|---|---|---|---|
| | Yes | No | Yes | No | Yes | No |
| **Antihypertensive medication** | | | | | | |
| True-positive, N | 289 | 174 | 78 | 384 | 232 | 231 |
| True-negative, N | 484 | 125 | 193 | 420 | 244 | 365 |
| False-positive, N | 14 | 6 | 3 | 17 | 5 | 12 |
| False-negative, N | 17 | 11 | 3 | 25 | 8 | 20 |
| Sensitivity (95% CI) | 0.94 (0.92–0.97) | 0.94 (0.91–0.97) | 0.96 (0.92–1.00) | 0.94 (0.92–0.96) | 0.97 (0.94–0.99) | 0.92 (0.89–0.95) |
| Specificity (95% CI) | 0.97 (0.96–0.99) | 0.95 (0.92–0.99) | 0.98 (0.97–1.00) | 0.96 (0.94–0.98) | 0.98 (0.96–1.00) | 0.97 (0.95–0.99) |
| Kappa score (95% CI) | 0.92 (0.89–0.95) | 0.89 (0.84–0.94) | 0.95 (0.91–0.99) | 0.90 (0.87–0.93) | 0.95 (0.92–0.98) | 0.89 (0.86–0.93) |
| **Diabetes medication** | | | | | | |
| True-positive, N | 78 | 47 | 22 | 104 | 46 | 79 |
| True-negative, N | 708 | 253 | 249 | 714 | 432 | 527 |
| False-positive, N | 12 | 11 | 3 | 20 | 8 | 14 |
| False-negative, N | 6 | 5 | 3 | 8 | 3 | 8 |
| Sensitivity (95% CI) | 0.93 (0.87–0.98) | 0.90 (0.82–0.98) | 0.88 (0.75–1.01) | 0.93 (0.88–0.98) | 0.94 (0.87–1.01) | 0.91 (0.85–0.97) |
| Specificity (95% CI) | 0.98 (0.97–0.99) | 0.96 (0.93–0.98) | 0.99 (0.97–1.00) | 0.97 (0.96–0.98) | 0.98 (0.97–0.99) | 0.97 (0.96–0.99) |
| Kappa score (95% CI) | 0.88 (0.83–0.94) | 0.82 (0.74–0.91) | 0.87 (0.76–0.97) | 0.86 (0.81–0.91) | 0.88 (0.81–0.95) | 0.86 (0.80–0.92) |
| **Dyslipidemia medication** | | | | | | |
| True-positive, N | 126 | 69 | 22 | 174 | 77 | 119 |
| True-negative, N | 618 | 212 | 237 | 595 | 375 | 451 |
| False-positive, N | 8 | 6 | 3 | 11 | 5 | 9 |
| False-negative, N | 52 | 29 | 15 | 66 | 32 | 49 |
| Sensitivity (95% CI) | 0.71 (0.64–0.77) | 0.70 (0.61–0.79) | 0.59 (0.44–0.75) | 0.73 (0.67–0.78) | 0.71 (0.62–0.79) | 0.71 (0.64–0.78) |
| Specificity (95% CI) | 0.99 (0.98–1.00) | 0.97 (0.95–0.99) | 0.99 (0.97–1.00) | 0.98 (0.97–0.99) | 0.99 (0.98–1.00) | 0.98 (0.97–0.99) |
| Kappa score (95% CI) | 0.76 (0.71–0.82) | 0.72 (0.64–0.81) | 0.67 (0.54–0.81) | 0.76 (0.71–0.81) | 0.76 (0.69–0.83) | 0.75 (0.68–0.81) |

CI, confidence interval.

**eTable 7.** Validity of self-reported medication use among subgroups with a 3-month fixed time window in males (continued)

| | Regular exerciser | |
|---|---|---|
| | Yes | No |
| **Antihypertensive medication** | | |
| True-positive, N | 166 | 297 |
| True-negative, N | 217 | 392 |
| False-positive, N | 9 | 8 |
| False-negative, N | 9 | 19 |
| Sensitivity (95% CI) | 0.95 (0.92–0.98) | 0.94 (0.91–0.97) |
| Specificity (95% CI) | 0.96 (0.93–0.99) | 0.98 (0.97–0.99) |
| Kappa score (95% CI) | 0.91 (0.87–0.95) | 0.92 (0.89–0.95) |
| **Diabetes medication** | | |
| True-positive, N | 31 | 94 |
| True-negative, N | 356 | 603 |
| False-positive, N | 9 | 13 |
| False-negative, N | 5 | 6 |
| Sensitivity (95% CI) | 0.86 (0.75–0.97) | 0.94 (0.89–0.99) |
| Specificity (95% CI) | 0.98 (0.96–0.99) | 0.98 (0.98–0.99) |
| Kappa score (95% CI) | 0.80 (0.69–0.90) | 0.89 (0.85–0.94) |
| **Dyslipidemia medication** | | |
| True-positive, N | 69 | 127 |
| True-negative, N | 297 | 529 |
| False-positive, N | 6 | 8 |
| False-negative, N | 29 | 52 |
| Sensitivity (95% CI) | 0.70 (0.61–0.79) | 0.64 (0.64–0.78) |
| Specificity (95% CI) | 0.98 (0.96–1.00) | 0.99 (0.97–1.00) |
| Kappa score (95% CI) | 0.74 (0.66–0.82) | 0.76 (0.70–0.81) |

CI, confidence interval.

**eTable 8.** Validity of self-reported medication-use among subgroups with a 3-month fixed time window in females

| | Age | | Married | | More than 12 years of education | |
|---|---|---|---|---|---|---|
| | 65–78 years | 35–64 years | Yes | No | Yes | No |
| **Antihypertensive medication** | | | | | | |
| True-positive, N | 346 | 102 | 348 | 98 | 82 | 365 |
| True-negative, N | 462 | 388 | 674 | 171 | 184 | 662 |
| False-positive, N | 20 | 4 | 19 | 5 | 4 | 19 |
| False-negative, N | 19 | 3 | 18 | 4 | 2 | 19 |
| Sensitivity (95% CI) | 0.95 (0.93–0.97) | 0.97 (0.94–1.00) | 0.95 (0.93–0.97) | 0.96 (0.92–1.00) | 0.98 (0.94–1.01) | 0.95 (0.93–0.97) |
| Specificity (95% CI) | 0.96 (0.94–0.98) | 0.99 (0.98–1.00) | 0.97 (0.96–0.98) | 0.97 (0.95–1.00) | 0.98 (0.96–1.00) | 0.97 (0.96–0.98) |
| Kappa score (95% CI) | 0.91 (0.88–0.93) | 0.96 (0.93–0.99) | 0.92 (0.90–0.95) | 0.93 (0.89–0.98) | 0.95 (0.91–0.99) | 0.92 (0.90–0.95) |
| **Diabetes medication** | | | | | | |
| True-positive, N | 63 | 22 | 65 | 19 | 10 | 74 |
| True-negative, N | 772 | 473 | 985 | 255 | 261 | 979 |
| False-positive, N | 10 | 1 | 7 | 3 | 1 | 9 |
| False-negative, N | 2 | 1 | 2 | 1 | 0 | 3 |
| Sensitivity (95% CI) | 0.97 (0.93–1.01) | 0.96 (0.87–1.04) | 0.97 (0.93–1.01) | 0.95 (0.85–1.05) | 1.00 (–) | 0.96 (0.92–1.00) |
| Specificity (95% CI) | 0.99 (0.98–1.00) | 1.00 (0.99–1.00) | 0.99 (0.99–1.00) | 0.99 (0.98–1.00) | 1.00 (0.99–1.00) | 0.99 (0.98–1.00) |
| Kappa score (95% CI) | 0.91 (0.85–0.96) | 0.95 (0.89–1.00) | 0.93 (0.89–0.98) | 0.90 (0.80–1.00) | 0.95 (0.85–1.00) | 0.92 (0.87–0.96) |
| **Dyslipidemia medication** | | | | | | |
| True-positive, N | 338 | 108 | 346 | 96 | 81 | 363 |
| True-negative, N | 466 | 375 | 670 | 168 | 184 | 652 |
| False-positive, N | 16 | 3 | 15 | 4 | 3 | 16 |
| False-negative, N | 27 | 11 | 28 | 10 | 4 | 34 |
| Sensitivity (95% CI) | 0.93 (0.90–0.95) | 0.91 (0.86–0.96) | 0.93 (0.90–0.95) | 0.91 (0.85–0.96) | 0.95 (0.91–1.00) | 0.91 (0.89–0.94) |
| Specificity (95% CI) | 0.97 (0.95–0.98) | 0.99 (0.98–1.00) | 0.98 (0.97–0.99) | 0.98 (0.95–1.00) | 0.98 (0.97–1.00) | 0.98 (0.96–0.99) |
| Kappa score (95% CI) | 0.90 (0.87–0.93) | 0.92 (0.88–0.96) | 0.91 (0.88–0.94) | 0.89 (0.84–0.95) | 0.94 (0.90–0.98) | 0.90 (0.87–0.93) |

CI, confidence interval.

**eTable 8.** Validity of self-reported medication-use among subgroups with a 3-month fixed time window in females (continued)

| | Currently working | | Current smoker | | Current drinker | |
|---|---|---|---|---|---|---|
| | Yes | No | Yes | No | Yes | No |
| **Antihypertensive medication** | | | | | | |
| True-positive, N | 210 | 238 | 8 | 437 | 15 | 433 |
| True-negative, N | 469 | 378 | 19 | 827 | 35 | 814 |
| False-positive, N | 9 | 15 | 0 | 24 | 0 | 24 |
| False-negative, N | 10 | 11 | 1 | 21 | 0 | 22 |
| Sensitivity (95% CI) | 0.95 (0.93–0.98) | 0.96 (0.93–0.98) | 0.89 (0.68–1.09) | 0.95 (0.93–0.97) | 1.00 (–) | 0.95 (0.93–0.97) |
| Specificity (95% CI) | 0.98 (0.97–0.99) | 0.96 (0.94–0.98) | 1.00 (–) | 0.97 (0.96–0.98) | 1.00 (–) | 0.97 (0.96–0.98) |
| Kappa score (95% CI) | 0.94 (0.91–0.96) | 0.92 (0.88–0.95) | 0.92 (0.75–1.00) | 0.92 (0.90–0.95) | 1.00 (–) | 0.92 (0.90–0.94) |
| **Diabetes medication** | | | | | | |
| True-positive, N | 48 | 36 | 1 | 84 | 1 | 84 |
| True-negative, N | 645 | 598 | 26 | 1212 | 47 | 1197 |
| False-positive, N | 3 | 7 | 1 | 10 | 2 | 9 |
| False-negative, N | 2 | 1 | 0 | 3 | 0 | 3 |
| Sensitivity (95% CI) | 0.96 (0.91–1.01) | 0.97 (0.92–1.03) | 1.00 (–) | 0.97 (0.93–1.00) | 1.00 (–) | 0.97 (0.93–1.00) |
| Specificity (95% CI) | 1.00 (0.99–1.00) | 0.99 (0.98–1.00) | 0.96 (0.89–1.03) | 0.99 (0.99–1.00) | 0.96 (0.90–1.01) | 0.99 (0.99–1.00) |
| Kappa score (95% CI) | 0.95 (0.90–0.99) | 0.89 (0.82–0.97) | 0.65 (0.019–1.00) | 0.92 (0.88–0.96) | 0.48 (–0.12–1.00) | 0.93 (0.89–0.97) |
| **Dyslipidemia medication** | | | | | | |
| True-positive, N | 201 | 244 | 5 | 440 | 5 | 441 |
| True-negative, N | 472 | 366 | 21 | 814 | 41 | 799 |
| False-positive, N | 8 | 11 | 0 | 19 | 1 | 18 |
| False-negative, N | 17 | 21 | 2 | 36 | 3 | 35 |
| Sensitivity (95% CI) | 0.92 (0.89–0.96) | 0.92 (0.89–0.95) | 0.71 (0.38–1.05) | 0.92 (0.90–0.95) | 0.63 (0.29–0.96) | 0.93 (0.90–0.95) |
| Specificity (95% CI) | 0.98 (0.97–0.99) | 0.97 (0.95–0.99) | 1.00 (–) | 0.98 (0.97–0.99) | 0.98 (0.93–1.02) | 0.98 (0.97–0.99) |
| Kappa score (95% CI) | 0.92 (0.88–0.95) | 0.90 (0.86–0.93) | 0.79 (0.51–1.00) | 0.91 (0.88–0.93) | 0.67 (0.37–0.97) | 0.91 (0.89–0.93) |

CI, confidence interval.

**eTable 8.** Validity of self-reported medication-use among subgroups with a 3-month fixed time window in females (continued)

| | Regular exerciser | |
|---|---|---|
| | Yes | No |
| **Antihypertensive medication** | | |
| True-positive, N | 149 | 299 |
| True-negative, N | 262 | 587 |
| False-positive, N | 5 | 19 |
| False-negative, N | 11 | 11 |
| Sensitivity (95% CI) | 0.93 (0.89–0.97) | 0.96 (0.94–0.99) |
| Specificity (95% CI) | 0.98 (0.97–1.00) | 0.97 (0.95–0.98) |
| Kappa score (95% CI) | 0.92 (0.88–0.96) | 0.93 (0.90–0.95) |
| **Diabetes medication** | | |
| True-positive, N | 23 | 62 |
| True-negative, N | 396 | 848 |
| False-positive, N | 6 | 5 |
| False-negative, N | 2 | 1 |
| Sensitivity (95% CI) | 0.92 (0.81–1.03) | 0.98 (0.95–1.01) |
| Specificity (95% CI) | 0.99 (0.97–1.00) | 0.99 (0.99–1.00) |
| Kappa score (95% CI) | 0.84 (0.73–0.95) | 0.95 (0.91–0.99) |
| **Dyslipidemia medication** | | |
| True-positive, N | 158 | 288 |
| True-negative, N | 253 | 587 |
| False-positive, N | 3 | 16 |
| False-negative, N | 13 | 25 |
| Sensitivity (95% CI) | 0.92 (0.88–0.96) | 0.92 (0.89–0.95) |
| Specificity (95% CI) | 0.99 (0.98–1.00) | 0.97 (0.96–0.99) |
| Kappa score (95% CI) | 0.92 (0.88–0.96) | 0.90 (0.87–0.93) |

CI, confidence interval.

**eTable 9.** Odds ratios (with 95% confidence intervals) for individual determinants associated with failure to report regularly dispensed medications by sex

| | Antihypertensive medication | | Diabetes medication | | Dyslipidemia medication | |
|---|---|---|---|---|---|---|
| | OR (95% CI) | Adjusted OR (95% CI) | OR (95% CI) | Adjusted OR (95% CI) | OR (95% CI) | Adjusted OR (95% CI) |
| **Males** | | | | | | |
| Age | 1.33 (0.55–3.20) | 1.07 (0.41–2.80) | 1.20 (0.30–4.75) | 1.08 (0.22–5.34) | 1.57 (0.87–2.84) | 1.73 (0.90–3.34) |
| Married | 1.29 (0.29–5.61) | 1.19 (0.26–5.48) | 0.79 (0.09–6.88) | 0.55 (0.05–5.80) | 1.13 (0.45–2.79) | 0.98 (0.38–2.52) |
| More than 12 years education | NA (NA) | NA (NA) | 2.21 (0.54–9.12) | 2.44 (0.53–11.2) | **0.41 (0.19–0.93)** | **0.41 (0.18–0.93)** |
| Currently working | 0.93 (0.43–2.03) | 1.14 (0.48–2.71) | 0.72 (0.21–2.50) | 1.00 (0.23–4.36) | 0.98 (0.57–1.69) | 1.11 (0.61–2.04) |
| Current smoker | 0.59 (0.17–2.01) | 0.81 (0.23–2.83) | 1.77 (0.44–7.22) | 2.00 (0.42–9.11) | 1.80 (0.88–3.67) | 2.02 (0.96–4.25) |
| Current drinker | **0.40 (0.17–0.92)** | **0.41 (0.17–0.97)** | 0.64 (0.16–2.55) | 0.59 (0.13–2.61) | 1.01 (0.59–1.71) | 1.13 (0.65–1.97) |
| Regular exerciser | 0.85 (0.38–1.92) | 0.76 (0.31–1.82) | 2.53 (0.72–8.86) | 1.70 (0.41–7.01) | 1.03 (0.60–1.76) | 1.03 (0.58–1.83) |
| **Females** | | | | | | |
| Age | 1.87 (0.54–6.44) | 2.63 (0.57–12.1) | 0.70 (0.06–8.09) | 0.31 (0.02–6.00) | 0.78 (0.38–1.63) | 0.81 (0.36–1.83) |
| Married | 1.27 (0.42–3.83) | 1.86 (0.53–6.62) | 0.59 (0.05–6.80) | 0.67 (0.04–12.0) | 0.78 (0.37–1.66) | 0.87 (0.39–1.94) |
| More than 12 years education | 0.47 (0.11–2.05) | 0.54 (0.12–2.42) | NA (NA) | NA (NA) | 0.53 (0.18–1.53) | 0.44 (0.15–1.33) |
| Currently working | 1.03 (0.43–2.48) | 1.24 (0.49–3.11) | 1.50 (0.13–17.2) | 1.68 (0.11–25.8) | 0.98 (0.51–1.91) | 0.96 (0.46–1.97) |
| Current smoker | 2.60 (0.31–21.8) | 5.67 (0.55–58.5) | NA (NA) | NA (NA) | 4.89 (0.92–26.1) | 3.44 (0.50–23.7) |
| Current drinker | NA (NA) | NA (NA) | NA (NA) | NA (NA) | **7.56 (1.74–33.0)** | **7.26 (1.44–36.6)** |
| Regular exerciser | 2.01 (0.85–4.74) | 2.18 (0.88–5.38) | 5.39 (0.47–62.3) | 8.84 (0.58–136) | 0.95 (0.47–1.90) | 1.04 (0.50–2.14) |

CI, confidence interval; NA, not available due to zero false negatives; OR, odds ratio.

The following factors were used as controls: 35–64 years, no marital status, 12 or fewer years of education years, not currently working, non-current smoker, non-current drinker and non-regular exerciser.

**eTable 10.** Odds ratios for individual determinants associated with failure to report regularly dispensed medications by education years

| | Antihypertensive medication | | Diabetes medication | | Dyslipidemia medication | |
|---|---|---|---|---|---|---|
| | OR (95% CI) | Adjusted OR (95% CI) | OR (95% CI) | Adjusted OR (95% CI) | OR (95% CI) | Adjusted OR (95% CI) |
| **More than 12 years education** | | | | | | |
| Sex | NA (NA) | NA (NA) | NA (NA) | NA (NA) | **4.43 (2.15–9.12)** | 5.34 (0.98–29.2) |
| Age | NA (NA) | NA (NA) | 0.32 (0.03–4.01) | NA (NA) | 1.95 (0.50–7.55) | 1.21 (0.25–5.88) |
| Married | NA (NA) | NA (NA) | NA (NA) | NA (NA) | 0.66 (0.17–2.64) | 0.50 (0.10–2.54) |
| Currently working | NA (NA) | NA (NA) | NA (NA) | NA (NA) | 0.57 (0.17–1.89) | 0.43 (0.09–2.06) |
| Current smoker | NA (NA) | 0.46 (NA) | NA (NA) | NA (NA) | 2.85 (0.53–15.3) | 2.12 (0.33–13.7) |
| Current drinker | NA (NA) | 0.59 (NA) | 5.71 (0.45–73.2) | NA (NA) | 3.22 (0.93–11.1) | 1.21 (0.26–5.68) |
| Regular exerciser | 1.58 (0.10–25.6) | 5.50 (0.24–129) | 3.40 (0.27–42.4) | NA (NA) | 1.88 (0.57–6.19) | 1.96 (0.55–7.04) |
| **12 or fewer years of education years** | | | | | | |
| Sex | 1.44 (0.79–2.62) | 1.89 (0.97–3.71) | 1.58 (0.10–25.6) | 2.99 (0.66–13.5) | **5.03 (3.21–7.87)** | **4.19 (2.50–7.01)** |
| Age | 1.41 (0.67–2.97) | 1.25 (0.58–2.73) | 1.64 (0.34–7.85) | 1.01 (0.18–5.68) | 0.93 (0.58–1.51) | 1.25 (0.72–2.15) |
| Married | 1.49 (0.58–3.86) | 1.35 (0.51–3.55) | 0.68 (0.14–3.40) | 0.51 (0.09–2.92) | 1.45 (0.79–2.66) | 1.02 (0.54–1.94) |
| Currently working | 0.90 (0.50–1.62) | 1.05 (0.56–1.96) | 0.53 (0.16–1.80) | 0.73 (0.19–2.88) | 1.48 (0.97–2.26) | 1.10 (0.68–1.78) |
| Current smoker | 0.97 (0.34–2.80) | 1.11 (0.37–3.36) | NA (NA) | NA (NA) | **4.27 (2.09–8.70)** | **2.29 (1.07–4.91)** |
| Current drinker | 0.56 (0.26–1.22) | **0.41 (0.17–0.96)** | 0.35 (0.04–2.82) | 0.31 (0.03–2.77) | **2.97 (1.80–4.91)** | 1.22 (0.69–2.18) |
| Regular exerciser | 1.34 (0.73–2.45) | 1.21 (0.65–2.26) | 2.58 (0.75–8.85) | 2.01 (0.51–7.86) | 0.92 (0.59–1.43) | 0.93 (0.57–1.50) |

CI, confidence interval; NA, not available due to zero false negatives; OR, odds ratio.

The following factors were used as controls: female sex, 35-64 years, no marital status, not currently working, non-current smoker, non-current drinker and non-regular exerciser.

**eTable 11.** Odds ratios for individual determinants associated with failure to report regularly dispensed medications by smoking status

| | Antihypertensive medication | | Diabetes medication | | Dyslipidemia medication | |
|---|---|---|---|---|---|---|
| | OR (95% CI) | Adjusted OR (95% CI) | OR (95% CI) | Adjusted OR (95% CI) | OR (95% CI) | Adjusted OR (95% CI) |
| **Current smoker** | | | | | | |
| Sex | 0.31 (0.03–3.32) | NA (NA) | NA (NA) | NA (NA) | 1.70 (0.29–10.0) | 1.66 (0.10–27.4) |
| Age | 0.65 (0.09–4.87) | NA (NA) | 0.39 (0.03–4.87) | NA (NA) | 0.32 (0.09–1.14) | 0.29 (0.07–1.22) |
| Married | NA (NA) | NA (NA) | NA (NA) | NA (NA) | 0.58 (0.10–3.29) | 1.10 (0.06–20.4) |
| More than 12 years education | NA (NA) | NA (NA) | NA (NA) | NA (NA) | 0.32 (0.06–1.72) | 0.34 (0.05–2.21) |
| Currently working | 0.10 (0.00–0.98) | NA (NA) | NA (NA) | NA (NA) | 0.55 (0.13–2.27) | 0.41 (0.07–2.55) |
| Current drinker | 1.87 (0.19–18.7) | NA (NA) | 2.60 (0.21–32.9) | NA (NA) | 1.91 (0.56–6.55) | 1.87 (0.46–7.57) |
| Regular exerciser | NA (NA) | NA (NA) | 5.67 (0.43–74.4) | NA (NA) | 0.83 (0.22–3.10) | 0.75 (0.17–3.40) |
| **Non-current smoker** | | | | | | |
| Sex | 1.36 (0.75–2.46) | 1.92 (0.99–3.74) | 2.15 (0.55–8.37) | 2.99 (0.66–13.6) | **4.64 (2.98–7.22)** | **4.42 (2.66–7.33)** |
| Age | 1.63 (0.75–3.56) | 1.66 (0.71–3.91) | 1.68 (0.35–8.02) | 1.01 (0.18–5.68) | 1.57 (0.92–2.66) | 1.74 (0.97–3.13) |
| Married | 1.20 (0.50–2.89) | 1.33 (0.50–3.53) | 0.71 (0.14–3.54) | 0.51 (0.09–2.92) | 1.40 (0.77–2.56) | 1.01 (0.54–1.89) |
| More than 12 years education | **0.21 (0.05–0.87)** | **0.23 (0.06–0.97)** | NA (NA) | NA (NA) | **0.47 (0.24–0.94)** | **0.49 (0.24–0.99)** |
| Currently working | 1.24 (0.67–2.27) | 1.55 (0.81–2.96) | 0.60 (0.18–2.04) | 0.73 (0.19–2.88) | 1.27 (0.83–1.94) | 1.11 (0.69–1.79) |
| Current drinker | 0.40 (0.15–1.02) | **0.28 (0.10–0.78)** | 0.41 (0.05–3.27) | 0.31 (0.03–2.77) | **2.57 (1.55–4.28)** | 1.20 (0.66–2.16) |
| Regular exerciser | 1.43 (0.78–2.60) | 1.40 (0.74–2.65) | 2.41 (0.70–8.27) | 2.01 (0.51–7.86) | 1.05 (0.68–1.62) | 1.10 (0.63–1.61) |

CI, confidence interval; NA, not available due to zero false negatives; OR, odds ratio.

The following factors were used as controls: female sex, 35-64 years, no marital status, 12 or fewer years of education years, not currently working, non-current drinker and non-regular exerciser.

**eTable 12.** Characteristics of the study population by concordance and discordance groups between health insurance claims and self-reported medication use

| | Total | Antihypertensive medication | | | Diabetes medication | | | Dyslipidemia medication | | |
|---|---|---|---|---|---|---|---|---|---|---|
| | | Concordance group | Discordance group | | Concordance group | Discordance group | | Concordance group | Discordance group | |
| | N (%) | N (%) | N (%) | *P*-value [a] | N (%) | N (%) | *P*-value [a] | N (%) | N (%) | *P*-value [a] |
| N | 2,472 | 2,378 | 94 | | 2,424 | 48 | | 2,320 | 152 | |
| Sex | | | | 0.2809 | | | **0.0004** | | | **<0.0001** |
| Male | 1,128 (45.6 %) | 1,080 (45.4 %) | 48 (51.1 %) | | 1,094 (45.1%) | 34 (70.8%) | | 1,033 (44.5%) | 95 (62.5%) | |
| Female | 1,344 (54.4 %) | 1,298 (54.6 %) | 46 (48.9 %) | | 1,330 (54.9%) | 14 (29.2%) | | 1,287 (55.5%) | 57 (37.5%) | |
| Age (65-78 years), Yes | 1,520 (61.5 %) | 1,445 (60.8 %) | 75 (79.8 %) | **0.0002** | 1483 (61.2%) | 37 (77.1%) | **0.0250** | 1,406 (60.6%) | 114 (75.0%) | **0.0004** |
| Married, Yes | 2,057 (83.2 %) | 1,978 (83.2 %) | 79 (84.0 %) | 0.7507 | 2,019 (83.3%) | 38 (79.2%) | 0.8316 | 1,929 (83.1%) | 128 (84.2%) | 0.7273 |
| Education years over 12 years, Yes | 509 (20.6 %) | 501 (21.1 %) | 8 (8.51 %) | **0.0038** | 502 (20.7%) | 7 (14.6%) | 0.3212 | 487 (21.0%) | 22 (14.5%) | 0.0505 |
| Currently working, Yes | 1,502 (60.8 %) | 1,452 (61.1 %) | 50 (53.2 %) | 0.1414 | 1,479 (61.0%) | 23 (47.9%) | 0.0853 | 1,417 (61.1%) | 85 (55.9%) | 0.1800 |
| Current smoker, Yes | 305 (12.3 %) | 298 (12.5 %) | 7 (7.45 %) | 0.1375 | 298 (12.3%) | 7 (14.6%) | 0.6427 | 285 (12.3%) | 20 (13.2%) | 0.7693 |
| Current drinker, Yes | 539 (21.8 %) | 526 (22.1 %) | 13 (13.8 %) | 0.0731 | 526 (21.7%) | 13 (27.1%) | 0.3360 | 498 (21.5%) | 41 (27.0%) | 0.1192 |
| Regular exerciser, Yes | 828 (33.5 %) | 794 (33.4 %) | 34 (36.2 %) | 0.4461 | 806 (33.3%) | 22 (45.8%) | 0.0541 | 777 (33.5%) | 51 (33.6%) | 0.9772 |

[a] *P*-values for differences between concordance and discordance groups were determined by using Chi-square test.

**eMaterials 1.** The standardized self-administrated questionnaire which was used in the Tsuruoka Metabolomics Cohort Study

定期的（週に1回以上）に飲んでいる薬がありますか？（サプリメントは除きます）1

① ない

② ある → ・どんな薬ですか？（あてはまる□を全てチェック）

□11 血圧を下げる薬

□12 コレステロールを下げる(高脂血症)薬

□13 心筋梗塞・狭心症の薬

□14 血糖値を下げる(糖尿病)薬

□15 抗リウマチ薬

□16 骨を強くする薬・注射

□17 便秘薬

□18 胃薬

□19 抗不安薬・抗うつ薬

□20 もの忘れを改善する薬

□21 ぜんそくの薬

□22 抗アレルギー薬(花粉症の薬など)

□23 血を固まりにくくする(血液の流れをよくする)薬

□24 ステロイド

□25 甲状腺の薬

□26 痛み止め・解熱剤

□27 痛風の（尿酸値を下げる）薬

□28 眠るための薬 □29 その他（ ）30
