# Supplementary material for: Erratum to “Validity Assessment of Self-reported Medication Use for Hypertension, Diabetes, and Dyslipidemia in a Pharmacoepidemiologic Study by Comparison With Health Insurance Claims”
Source: J Epidemiol. 2021 Sep 5;31(9):520–1. doi: 10.2188/jea.JE20210109 (PMC8328859; doi:10.2188/jea.JE20210109)
Supplement: Supplementary file 1 [file je-31-520-s001.pdf]

eTable 1. Medications for hypertension

Therapeutic category of drugs in Japan		Generic name	ATC codes	
21: Cardiovascular agents	212: Antiarrhythmic agents	Propranolol hydrochloride	C07AA05	
		Bufetolol hydrochloride	C07	
		Nadolol	C07AA12	
		Pindolol	C07AA03	
		Carteolol hydrochloride	C07AA15	S01ED05
		Metoprolol tartrate	C07AB02	
		Atenolol	C07AB03	
		Bisoprolol	C07AB07	
		Betaxolol hydrochloride	C07AB05	S01ED02
		Acebutolol hydrochloride	C07AB04	
		Celiprolol hydrochloride	C07AB08	
		Labetalol hydrochloride	C07AG01	
		Carvedilol	C07AG02	
		Bevantolol hydrochloride	C07AB06	
		Amosulalol hydrochloride	C07	
		Arotinolol hydrochloride	C07	
		Nipradilol	C07	
	213: Diuretics	Trichlormethiazide	C03AA06	
		Hydrochlorothiazide	C03AA03	
		Benzylhydrochlorothiazide	C03	
		Indapamide	C03BA11	
		Tripamide	C03	
		Meticrane	C03BA09	
		Mefruside	C03BA05	
		Furosemide	C03CA01	
		Bumetanide	C03CA02	
		Azosemide	C03CA	
		Torasemide	C03CA04	
		Spironolactone	C03DA01	
		Triamterene	C03DB02	
21: Cardiovascular agents	214: Hypertensions	Captopril	C09AA01	
		Enalapril maleate	C09AA02	
		Alacepril	C09AA	
		Delapril hydrochloride	C09AA12	
		Cilazapril hydrate	C09AA08	
		Lisinopril hydrate	C09AA03	

eTable 1. Medications for hypertension (continued)

Therapeutic category of drugs in Japan	Generic name	ATC codes
	Benazepril hydrochloride	C09AA07
	Imidapril hydrochloride	C09AA16
	Temocapril hydrochloride	C09AA14
	Quinapril hydrochloride	C09AA06
	Trandolapril	C09AA10
	Perindopril erbumine	C09AA04
	Candesartan cilexetil	C09CA06
	Losartan potassium	C09CA01
	Valsartan	C09CA03
	Telmisartan	C09CA07
	Olmesartan medoxomil	C09CA08
	Irbesartan	C09CA04
	Azilsartan	C09CA09
	Losartan potassium・hydrochlorothiazide	C09DA01
	Candesartan cilexetil・hydrochlorothiazide	C09DA06
	Valsartan・hydrochlorothiazide	C09DA03
	Telmisartan・hydrochlorothiazide	C09DA07
	Irbesartan・trichlormethiazide	C09DA04
	Olmesartan medoxomil・azelnidipine	C09DB
	Valsartan・cilnidipine	C09DB
	Valsartan・amlodipine besilate	C09DB01
	Candesartan cilexetil・amlodipine besilate	C09DB07
	Azilsartan・amlodipine besilate	C09DB
	Telmisartan・amlodipine besilate	C09DB04
	Irbesartan・amlodipine besilate	C09DB05
	Telmisartan・amlodipine besilate・hydrochlorothiazide	C09DX
	Aliskiren fumarate	C09XA02
	Eplerenone	C03DA04
	Prazosin hydrochloride	C02CA01

eTable 1. Medications for hypertension (continued)

Therapeutic category of drugs in Japan	Generic name	ATC codes	
	Bunazosin hydrochloride	C02	
	Terazosin hydrochloride hydrate	C02	G04CA03
	Urapidil	C02CA06	
	Doxazosin mesilate	C02CA04	
	Clonidine hydrochloride	C02AC01	N02CX02
	Methyldopa hydrate	C02AB01	
	Guanabenz acetat	C02A	
	Reserpine	C02AA02	
	Hydralazine hydrochloride	C02DB02	
	Benzylhydrochlorothiazide・reserpine combinations	C02AA52	C02LA51
	Ambrisentan	C02KX02	
	Selexipag	B01AC27	C02
	Tadalafil	G04BE08	C02
	Sildenafil citrate	G04BE03	C02
	Macitentan	C02KX04	
	Riociguat	C02KX05	
	Bosentan hydrate	C02KX01	
	Beraprost sodium	B01AC19	C02
	Nifedipine	C08CA05	
	Nicardipine hydrochloride	C08CA04	
	Nilvadipine	C08CA10	
	Manidipine hydrochloride	C08CA11	
	Barnidipine hydrochloride	C08CA12	
	Efonidipine hydrochloride ethanolate	C08	
	Felodipine	C08CA02	
	Cilnidipine	C08CA14	
	Aranidipine	C08	
	Verapamil hydrochloride	C08DA01	
	Azelnidipine	C08	
217: Vasodilators	Amlodipine besilate	C08CA01	
	Nisoldipine	C08CA07	
	Nitrendipine	C08CA08	
	Diltiazem hydrochloride	C05AE03	C08DB01
	Benidipine hydrochloride	C08CA15	
219: Miscellaneous	Atorvastatin calcium hydrate・amlodipine besilate	C10BX03	

ATC, Anatomical Therapeutic Chemical.

eTable 2. Medications for diabetes

Therapeutic category of drugs in Japan		Generic name	ATC codes
39: Other agents affecting metabolism	396: Antidiabetic agents	Chlorpropamide	A10BB02
		Acetohexamide	A10BB31
		Glyclopyramide	A10BB
		Glibenclamide	A10BB01
		Gliclazide	A10BB09
		Glimepiride	A10BB12
		Tolbutamide	A10BB03
		Buformin hydrochloride	A10BA03
		Metformin hydrochloride	A10BA02
		Pioglitazone hydrochloride	A10BG03
		Acarbose	A10BF01
		Voglibose	A10BF03
		Miglitol	A10BF02
		Nateglinide	A10BX03
		Mitiglinide calcium hydrate	A10BX08
		Repaglinide	A10BX02
		Sitagliptin phosphate hydrate	A10BH01
		Vildagliptin	A10BH02
		Alogliptin benzoate	A10BH04
		Linagliptin	A10BH05
		Teneligliptin hydrobromide hydrate	A10BH08
		Anagliptin	A10BH
		Saxagliptin hydrate	A10BH03
		Trelagliptin succinate	A10BH
		Omarigliptin	A10BH
		Ipragliflozin L-proline	A10BK05
		Tofogliflozin hydrate	A10BK
		Dapagliflozin propylene glycolate hydrate	A10BK01
		Luseogliflozin hydrate	A10BK07
		Canagliflozin hydrate	A10BK02
		Empagliflozin	A10BK03

eTable 2. Medications for diabetes (continued)

Therapeutic category of drugs in Japan	Generic name	ATC codes
	Metformin hydrochloride・pioglitazone hydrochloride	A10BD05
	Metformin hydrochloride・vildagliptin	A10BD08
	Glimepiride・pioglitazone hydrochloride	A10BD06
	Mitiglinide calcium hydrate・voglibose	A10BD
	Pioglitazone hydrochloride・alogliptin benzoate	A10BD09
	Metformin hydrochloride・alogliptin benzoate	A10BD13
	Teneligliptin hydrobromide hydrate・canagliflozin hydrate	A10BD
	Sitagliptin phosphate hydrate・ipragliflozin L-proline	A10BD

ATC, Anatomical Therapeutic Chemical.

eTable 3. Medications for dyslipidemia

Therapeutic category of drugs in Japan		Generic name	ATC codes	
21: Cardiovascular agents	218: Hyperlipidemia agents	Pravastatin sodium	C10AA03	
		Simvastatin	C10AA01	
		Fluvastatin sodium	C10AA04	
		Atorvastatin calcium hydrate	C10AA05	
		Pitavastatin calcium hydrate	C10AA08	
		Rosuvastatin calcium	C10AA07	
		Atorvastatin calcium hydrate・amlodipine besilate	C10BX03	
		Clofibrate	C10AB01	
		Clinofibrate	C10	
		Bezafibrate	C10AB02	
		Fenofibrate	C10AB05	
		Pemafibrate	C10	
		Ezetimibe	C10AX09	
		Atorvastatin calcium hydrate・ezetimibe	C10BA05	
		Lomitapide mesilate	C10AX12	
		Nicomol	C10	
		Niceritrol	C10AD01	
		Colestyramine	C10AC01	
		Colestimide	C10AC	V03AE06
		Probucol	C10AX02	
		Gamma oryzanol	C10	
		Dextran sulfate sodium sulfur 18	C10	B05AA05
		Polyenephosphatidyl choline	C10	
		Elastase ES	C10	
		Ethyl icosapentate	C10	
		Omega-three-acid ethyl esters	C10AX	

ATC, Anatomical Therapeutic Chemical.
